# Polyamidoamine-Carbon Nanodot Conjugates with Bioreducible Building Blocks: Smart Theranostic Platforms for Targeted siRNA Delivery

**DOI:** 10.1021/acs.biomac.3c01185

**Published:** 2024-01-05

**Authors:** Salvatore Emanuele Drago, Mara Andrea Utzeri, Nicolò Mauro, Gennara Cavallaro

**Affiliations:** Laboratory of Biocompatible Polymers, Department of Biological, Chemical and Pharmaceutical Sciences and Technologies (STEBICEF), University of Palermo, Via Archirafi 32, 90123 Palermo, Italy

## Abstract

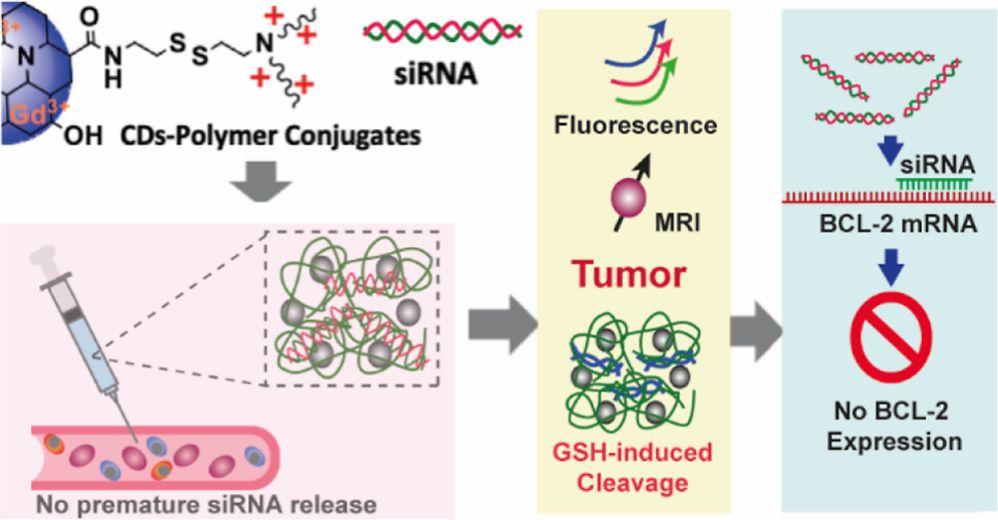

This study focuses on designing hybrid theranostic nanosystems, utilizing gadolinium-doped carbon nanodots decorated with bioreducible amphoteric polyamidoamines (PAAs). The objective is to synergize the exceptional theranostic properties of gadolinium-doped carbon nanodots (CDs) with the siRNA complexation capabilities of PAAs. Linear copolymeric polyamidoamines, based on *N*,*N*′-bis(acryloyl)cystamine, arginine, and agmatine, were synthesized, resulting in three distinct amphoteric copolymers. Notably, sulfur bridges within the PAA repeating units confer pronounced susceptibility to glutathione-mediated degradation—a key attribute in the tumor microenvironment. This pathway enables controlled and stimuli-responsive siRNA release, theoretically providing precise spatiotemporal control over therapeutic interventions. The selected PAA, conjugated with CDs using the redox-sensitive spacer cystamine, formed the CDs-Cys-PAA conjugate with superior siRNA complexing capacity. Stable against polyanion exchange, the CDs-Cys-PAA/siRNA complex released siRNA in the presence of GSH. In vitro studies assessed cytocompatibility, internalization, and gene silencing efficacy on HeLa, MCF-7, and 16HBE cell lines.

## Introduction

Recently, considerable efforts have been made to identify different cancer genes and their communication pathways in order to guide the development of siRNA-mediated therapies for cancer treatment.^1−3^ There are several examples of siRNAs specifically designed to target oncogenes such as Bcl-2,^4^ Kirsten’s sarcoma,^5^ and c-myc.^6^ However, although siRNA-based therapies have great therapeutic potential, several obstacles limit their effectiveness as new treatments^2^ due to its chemical–physical properties.^7−10^ For instance, although naked siRNAs can efficiently silence genes in vitro, they are usually unstable; being negatively charged, they are membrane-impermeable and are typically degraded after administration in humans due to the vulnerability of the phosphodiester bond of siRNA by RNases and phosphatases. Thus, once they are administrated in vivo into the bloodstream, nucleases will quickly degrade siRNA into fragments, preventing the accumulation of therapeutic siRNA in the tumor.^11^ Hence, the design and development of efficient siRNA delivery systems that are both protective and able to deliver siRNA to the site of action remain at the forefront of research endeavors.

Despite numerous examples where viral vectors have proven to be valuable tools for siRNA delivery, the issues associated with viral vectors, such as, for example, the high immunogenicity and potential host immune responses, have led scientists to develop nonviral systems, which are considered to be much safer than viral systems.^12,13^

On this ground, polymers offer numerous advantages over nonviral vectors, as they can load higher amount of oligonucleotides and can be chemically modified to produce systems tailored to target specific tumors.^14^ Among them, amphoteric poly(amidoamine)s (PAAs) are a class of biocompatible and biodegradable synthetic polymers that are of great interest due to their high transfection efficiency for siRNA.^15−18^ PAAs are obtained by aza-Michael polyaddition between primary or bis-secondary aliphatic amines and bis(acrylamide)s. Depending on the choice of monomer precursors used in the polymerization, PAAs can be designed to carry different functional groups in the side chain.^19−21^ Highly biocompatible self-buffered PAAs with a prevailingly cationic behavior at physiological pH can be easily obtained using amino acids such as arginine as monomer precursors.^22−24^ Besides, agmatine-bearing, linear PAAs are efficient siRNA condensing agents and, if partially counterbalanced by anionic charges such as carboxylic acid moieties, exhibit higher biocompatibility than polycations.^25,26^ Therefore, the versatility of the synthetic process allows the obtaining of modular and multifunctional polyampholytes that combine adequate complexing capacity with high cytocompatibility, unlike other cationic polymers proposed for gene delivery (i.e., b-PEI, l-PEI, etc.) which are characterized by considerable toxicity.^27^ It is noteworthy that PAAs containing disulfide bonds in their repeating units have gained considerable interest due to their exceptional potential for the delivery of active compounds within the tumor microenvironment (TME). This is attributed to their ability to undergo glutathione-mediated reduction, facilitating their biodegradation into short fragments and the controlled release of their payload in a stimuli-responsive manner.^28^

While PAAs demonstrate great potential as siRNA complexing agents, achieving theranostic polyplexes able to simultaneously act as a therapeutic and diagnostic tool is not feasible. Indeed, for a system to serve both as an antitumor agent and as a cancer cell tracker for tumor diagnosis and monitoring, it is crucial that it may be remotely traced in a noninvasive manner, such as by operating as a contrast agent in fluorescence imaging (FLI), magnetic resonance imaging (MRI), computed tomography (CT), and other modalities.^29−32^ Among nanotheranostic systems with the most promising features, carbon nanodots (CDs) have shown many attractive properties such as tunable photoluminescence (PL), light-induced photothermal conversion, strong hydrophilicity, and high biocompatibility.^33,34^ In addition, they can be doped with paramagnetic ions such as gadolinium to combine multimodal imaging (i.e., FLI and MRI contrast) useful for image-guided anticancer drug release applications.^29,35−37^ Moreover, their surface functional groups enhance aqueous solubility and provide flexibility in surface chemical modifications, such as conjugation with small molecules or functional macromolecules.^38−40^ Nevertheless, modifying the surface of CDs to make it suitable for siRNA delivery while maintaining their ultrasmall dimensions, optical properties, good biocompatibility, and dispersibility in aqueous media is not a trivial task. Indeed, surface engineering of CDs with polycations can compromise their cytocompatibility and quench their typical fluorescence owing to perturbation of electronic states at the surface.^41,42^

In this context, we centered our attention on designing hybrid theranostic nanosystems, employing gadolinium-doped carbon nanodots, hereafter referred to as CDs, strategically functionalized at the surface with disulfur-containing bioreducible amphoteric PAAs, prevailingly cationic under physiological pH conditions. We harness the outstanding bimodal imaging attributes of CDs (i.e., fluorescence and MRI) and seamlessly integrate them with the promising siRNA complexation capabilities of PAAs, creating a singular, biodegradable, and totally bioeliminable nanoplatform.^29^ Specifically, we synthesized amphoteric and bioreducible PAAs, striving to attain an optimal equilibrium between siRNA complexing capacity and cytocompatibility. To this end, we synthesized three distinct linear copolymeric PAAs, employing *N*,*N*′-bis(acryloyl)cystamine (BAC), arginine, and agmatine as building blocks, followed by comprehensive characterization. The most promising PAA candidate was subsequently conjugated to the surface of CDs by a cystamine spacer, yielding the CDs-Cyst-PAA conjugate. Consequently, we evaluated the potential of this novel conjugate as a theranostic agent capable of effectively complexing and delivering a specific siRNA in a glutathione-dependent fashion. Additionally, we investigate its capacity to be internalized by cancer cells and induce gene silencing.

## Materials and Methods

### Materials

Agarose, agmatine sulfate, albumin, arginine, cystamine dichlorhydrate, 4′,6′-diamidine-2-phenylindole (DAPI), dimethylformamide anhydrous (DMFa), glutathione, lithium hydroxide, morpholine, *N*-(3-(dimethylamino)propyl)-*N*-ethylcarbodiimide hydrochloride (EDC-HCl), *N*-hydroxysuccinimide (NHS), *N*,*N*′-bis(acryloyl)cystamine, and Dulbecco’s phosphate buffered saline (DPBS) were purchased from Merck (Italy). SpectaPor dialysis membranes were purchased from Carlo Erba Reagent (Italy). Dulbecco’s Minimum Essential Medium (DMEM), fetal bovine serum (FBS), l-glutamine, penicillin, streptomycin, and amphotericin B were purchased from EuroClone (Milan, Italy). The CellTiter 96 AQueous One Solution Cell Proliferation Assay (MTS) was purchased from Promega (Milan, Italy). The siRNA duplex was purchased from QIAGEN (Milan, Italy). The sequences (5′ → 3′) are GGGAGAACAGGGUACGAUATT (sense), AAUAUCGUACCCUGUCCC (antisense). Gadolinium-doped CDs have been obtained as previously described.^29^ Salient characteristics of the CDs are reported in Figures S1 and S2 and the extensive characterization is reported elsewhere.^29^

### Methods

#### Cell Cultures

The following cell lines were used for biological characterization: immortalized normal bronchial epithelial cells (16-HBE) (provided by Istituto Zoo-profilattico di Lombardia ed Emilia Romagna), cervical cancer tumor cells (HeLa) (provided by Sigma-Aldrich), and breast cancer tumor cells (MCF-7) (provided by Sigma-Aldrich). Cells were maintained in a humidified atmosphere of 5% CO_2_ at 37 °C, cultured as adherent monolayers in DMEM supplemented with 10% FBS, 2 mM l-glutamine, 100 U/mL penicillin, 100 g/mL streptomycin, and 0.6 g/mL amphotericin B.

#### Nuclear Magnetic Resonance

^1^H NMR and ^13^C NMR spectra were recorded using a Bruker Avance II 400 spectrometer operating at 400.15 and 100.63 MHz, respectively.

#### Fourier-Transform Infrared Spectroscopy

Fourier-transform infrared spectroscopy (FT-IR) was performed in the range of 4000–500 cm^–1^. Samples were prepared as KBr pellets and dried in vacuum. Measurements were performed using a PerkinElmer Spectrum Two IR spectrometer (Waltham, MA, USA).

#### Size Exclusion Chromatography

Weighted molecular weight () and polydispersity (PD) of PAAs were evaluated by size exclusion chromatography (SEC). SEC analyses were performed with an Agilent 1260 Infinity instrument (Santa Clara, United States) equipped with a Yarra 2000 3 mm column connected in series to a Refractive Index (RI) detector. The  and PD were evaluated by relative calibration using standards of PEG ranging from 238 Da and 220 kDa. The mobile phase was a 0.1 M Tris buffer pH 8.2 with 0.2 M sodium chloride. The analyses were performed with a flow rate of 0.8 mL min^–1^ at 30 ± 1 °C.

#### Atomic Force Microscopy

AFM micrographs were obtained on a FAST-SCAN microscope equipped with a closed-loop scanner (*X*, *Y*, and *Z* maximum scan region: 35, 35, and 3 μm, respectively). The analysis was performed in soft tapping mode using a FAST-SCAN-A probe with an apical radius of 5 nm operating at 1400 kHz (*k*: 18 N/m).

### Synthesis of Cystamine-Functionalized CDs

Gd-doped CDs were functionalized with cystamine using *N*-(3-(dimethylamino)propyl)-*N*-ethylcarbodiimide hydrochloride (EDC-HCl), and *N*-hydroxysuccinimide (NHS) as coupling agents. CDs (40 mg) and cystamine hydrochloride (80 mg, 0.355 mmol) were solubilized in phosphate buffered saline (PBS) (3.8 mL) at pH 6.4 under stirring. The pH of the reaction was adjusted at 6.4 using 0.1 N NaOH and, then, the mixture of NHS (46.04 mg, 0.4 mmol) and EDC (170.2 mg, 0.8 mmol) in PBS pH 6.4 (200 μL) was added under stirring. The reaction was maintained at pH 6.4 for 24 h at room temperature and purified by SEC using a column packed in turn with Sephadex G-25 and G-15. The product was freeze-dried to get the cystamine-functionalized CDs (CDs-Cyst). Yield: 50%.

^1^H NMR CDs-Cyst (400 MHz, D_2_O, 25 °C, TMS): δ 3.0 (4H, NHCH_2_C**H**_**2**_SSC**H**_**2**_CH_2_NH_2_), 3.4 (4H, NHC**H**_**2**_CH_2_SSCH_2_C**H**_**2**_NH_2_).

### Synthesis of Amphoteric Bioreducible PAA Oligomers

PAA oligomers with different charge balances were obtained by the aza-Michael type polyaddition between l-arginine, agmatine, and BAC, by varying percentage ratios of l-arginine/agmatine monomers (i.e., 70:30, 50:50, and 30:70) and using excess bis-acrylamides. Typically, PAA oligomers were synthesized by solubilizing BAC (500 mg, 1.9 mmol) in water/DMF 2:1 (627 μL) and adding different amounts of arginine, agmatine, and lithium hydroxide as reported in Table 1.

**Table 1:** Chemical Composition of the Reaction Mixtures for PAA Synthesis

samples	BAC	arginine	agmatine sulfate	LiOH	water/DMF (2:1)
PAA_BAC_^[70:30]^	500 mg (1.9 mmol)	222 mg (1.3 mmol)	127 mg (0.5 mmol)	23 mg (0.5 mmol)	627 μL
PAA_BAC_^[50:50]^	500 mg (1.9 mmol)	159 mg (0.9 mmol)	212 mg (0.9 mmol)	38 mg (0.9 mmol)	627 μL
PAA_BAC_^[30:70]^	500 mg (1.9 mmol)	95 mg (0.5 mmol)	297 mg (1.3 mmol)	53 mg (1.3 mmol)	627 μL

The reaction was left at room temperature for 7 days under slow stirring. Under these conditions, the precipitation of oligomers occurred just after 3 days. Then, the reaction mixture was diluted with an equal volume of ultrapure water and the pH was adjusted to 3 with 37% v/v hydrochloric acid, yielding a yellowish solution. Products were purified by SEC using a column packed in turn with Sephadex G-25 and G-15. Then, solutions were filtered through a paper filter and freeze-dried to get white powders named PAA_BAC_^[70:30]^, PAA_BAC_^[50:50]^, and PAA_BAC_^[30:70]^. Yield: 48, 51, and 52%, respectively.

^1^H NMR PAA^[30:70]^ (400 MHz, D_2_O, 25 °C, TMS): δ 1.61 (4H, NHCH_2_C**H**_**2**_CH_2_CH_2_/NHCH_2_C**H**_**2**_CH_2_CH), 1.76–1.86 (4H, NHCH_2_CH_2_C**H**_**2**_CH_2_/NHCH_2_CH_2_C**H**_**2**_CH), 2.76 (8H, CH_2_C**H**_**2**_SS-C**H**_**2**_CH_2_), 2.82–2.84 (8H, C**H**_**2**_CH_2_NCH_2_C**H**_**2**_), 3.12–3.20 (8H, C**H**_**2**_CH_2_SSCH_2_C**H**_**2**_), 3.20–3.35 (6H, NHC**H**_**2**_CH_2_CH_2_C**H**_**2**_/NHC**H**_**2**_CH_2_CH_2_CH), 3.50–3.52 (8H, CH_2_C**H**_**2**_N–C**H**_**2**_CH_2_), 3.68–3.74 (1H, NHCH_2_CH_2_CH_2_C**H**), 5.72–5.75 (1H, C**H**_**2**_CH), 6.12–6.30 (2H, C**H**_**2**_C**H**).

^13^C NMR PAA^[30:70]^ (400 MHz, D_2_O, 25 °C, TMS): δ 21.2–32.0 (NHCH_2_**C**H_2_**C**H_2_CH_2_/NHCH_2_**C**H_2_**C**H_2_CH), 35.7 (**C**H_2_CH_2_NCH_2_**C**H_2_), 39.2 (CH_2_**C**H_2_SS-**C**H_2_CH_2_) 40,8 (**C**H_2_CH_2_SSCH_2_**C**H_2_), 44.1 (NH**C**H_2_CH_2_CH_2_**C**H_2_/NH**C**H_2_CH_2_CH_2_CH), 49.9 (CH_2_**C**H_2_N–**C**H_2_CH_2_), 52.3 (NHCH_2_CH_2_CH_2_**C**H_2_), 61.9 (NHCH_2_CH_2_CH_2_**C**H), 128.3 (CH**C**H_2_), 130.0 (**C**HCH_2_), 156.5 (NH_2_**C**NH), 164.2–168.1 (NH**C**CHCH_2_), 170.8 (NHCH_2_CH_2_CH_2_CH**C**OOH), 171.2 (NCH_2_CH_2_**C**NH).

### Evaluation of the Complexing Ability of PAA Oligomers toward a siRNA Model

Complexation studies were evaluated by gel retardation assay on 1.5% agarose gel with 0.1% ethidium bromide. In order to obtain different PAA/siRNA weight ratios (*R*) (i.e., *R* = 0.0, 1.6, 3, 6.25, 12.5, 25, or 50.0), polyplexes were prepared by adding PAA dispersions (10 μL) at different concentrations (0.16 to 5 mg mL^–1^) to the same volume of siRNA solution at a fixed concentration (10 μL, 0.1 mg mL^–1^). siRNA/PAA polyplexes (PAA@siRNA) were prepared in 10 mM nuclease-free Hepes buffer at pH 7.4 containing 5% (w/v) glucose. Mixtures were prepared by gently pipetting the solutions and incubating the samples for 30 min before the electrophoresis. For the electrophoretic mobility shift assay, each PAA@siRNA sample (10 μL) was placed on agarose gel and runs were performed in tris acetate/EDTA (TAE) buffer at pH 8.0 (100 V, 30 min). Naked siRNA was used as the positive control. The naked siRNA and polyplexes were visualized by using an UV trans-illuminator, collecting pictures by using a digital camera.

### Synthesis of the CDs-Cyst-PAA^[30:70]^ Conjugate

For the synthesis of the CDs-Cyst-PAA^[30:70]^ conjugate, an aqueous dispersion of CDs-Cyst at pH 10 (10 mg, 1.5 × 10^18^ nanoparticles, 30 μL) was added to a solution of PAA^[30:70]^ oligomers in water pH 10.0 (100 mg, 0.05 mmol, 30 × 10^20^ chains, 70 μL) under gentle stirring. The reaction mixture was maintained for 5 days at room temperature with occasional stirring. Then, the pH of the mixture was adjusted to 3.0 using 1 N hydrochloric acid and the product was purified by dialysis (MWCO 1 kDa) for 3 days. Then, the purified solution was filtered through a paper filter and freeze-dried, yielding a whitish powder (yield: 41%).

^1^H NMR CDs-Cyst-PAA^[30:70]^ (400 MHz, D_2_O, 25 °C, TMS): δ 1.63 (4H, NHCH_2_C**H**_**2**_CH_2_CH_2_/NHCH_2_–C**H**_**2**_CH_2_CH), 1.75–1.92 (4H, NHCH_2_CH_2_C**H**_**2**_CH_2_/NHCH_2_CH_2_C**H**_**2**_CH), 2.73 (16H, CH_2_C**H**_**2**_SSC**H**_**2**_CH_2_/CH_2_C**H**_**2**_SSC**H**_**2**_CH_2_NH), 2,85 (8H, C**H**_**2**_CH_2_NCH_2_C**H**_**2**_), 3.11 (8H, C**H**_**2**_CH_2_SSCH_2_C**H**_**2**_), 3.22–3.34 (6H, NHC**H**_**2**_CH_2_CH_2_C**H**_**2**_/NHC**H**_**2**_CH_2_CH_2_CH), 3.52 (8H, CH_2_C**H**_**2**_NC**H**_**2**_CH_2_), 3.68–3.74 (1H, NHCH_2_CH_2_CH_2_C**H**), 5.73–5.77 (1H, C**H**_**2**_CH), 6.15–6.32 (2H, C**H**_**2**_CH).

### Quenching of the CDs-Cyst-PAA^[30:70]^ End-Chains with Morpholine (CDs-Cyst-PAA^[30:70]^-M)

The double bond end-chains of CDs-Cyst-PAA^[30:70]^ were quenched by dispersing CDs-Cyst-PAA^[30:70]^ in water (20 mg, 0.4 g mL^–1^) and adding morpholine (6.5 mg; 7.5 × 10^–2^ mmol) dropwise under stirring. The reaction mixture was maintained at 25 °C for 24 h, the crude was purified from byproducts by dialysis (MWCO 1 kDa), and then freeze-dried to give a white powder (yield: 96%).

^1^H NMR CDs-Cys-PAA^[30:70]^-**M** (400 MHz, D_2_O, 25 °C, TMS): δ 1.58 (4H, NHCH_2_C**H**_**2**_CH_2_CH_2_/NHCH_2_–C**H**_**2**_CH_2_CH), 1.76–1.92 (4H, NHCH_2_CH_2_C**H**_**2**_CH_2_/NHCH_2_CH_2_C**H**_**2**_CH), 2.73 (16H, CH_2_C**H**_**2**_SSC**H**_**2**_CH_2_/CH_2_C**H**_**2**_SSC**H**_**2**_CH_2_NH), 2.80 (8H, C**H**_**2**_CH_2_NCH_2_C**H**_**2**_), 3.13 (4H, CH_2_C**H**_**2**_N C**H**_**2**_CH_2_O), 3,17 (8H, C**H**_**2**_CH_2_SSCH_2_C**H**_**2**_), 3.28–3.34 (6H, NHC**H**_**2**_CH_2_CH_2_C**H**_**2**_/NHC**H**_**2**_CH_2_CH_2_CH), 3.48 (8H, CH_2_C**H**_**2**_NC**H**_**2**_CH_2_), 3.88 (4H, C**H**_**2**_CH_2_N CH_2_C**H**_**2**_O), 4.11 (1H, NHCH_2_CH_2_CH_2_C**H**).

### Differential Scanning Calorimetry and Thermogravimetry of CDs-Cyst-PAA^[30:70]^-M

The percentage of PAA polymer chains bound on the CDs’ surface was evaluated by differential scanning calorimetry analysis coupled with thermogravimetric analysis (DSC-TGA). The analysis was conducted using a DSC/TGA 131 EVO (SETARAM Instr.) on about 2 mg of dried sample placed in an aluminum crucible under continuous flow of nitrogen (1 mL min^–1^). The temperature was ramped at a rate of 10 °C min^–1^ over the range of 30–600 °C. The thermograms were normalized by the sample weight and fitted as a function of the sample temperature.

### Spectrophotometric Determination of Gd^3+^ Ion Content in the CDs-Cyst-PAA^[30:70]^-M

The amount of Gd^3+^ ions in CDs-Cyst-PAA^[30:70]^-M was evaluated spectroscopically using xylenol orange (XO) tetrasodium salt as the metal indicator.^43^ CDs-Cyst-PAA^[30:70]^-M (7.2 mg) was mineralized using a microwave synthesizer Discover SP (CEM) by in turn treating it with 10% v/v HNO_3_ for 5 min at 100 °C and 15 min at 200 °C. At the end of the process, samples were cooled down and the pH adjusted to 5.8. A solution of XO in acetate buffer pH 5.8 (16 μg mL^–1^; 950 μL) was added to 50 μL of each sample. Then, the absorption spectrum was recorded within the range 400–600 nm and the amount of Gd^3+^ was calculated by comparing the 573/433 nm absorbance ratio against a calibration curve obtained with GdCl_3_ standards (10–30 μM) in acetate buffer pH 5.8 (*R*^2^ = 0.997). The Gd^3+^ loading was expressed as the weight percentage of Gd atoms entrapped in 100 mg of CDs-Cyst-PAA^[30:70]^-M.

### Optical Characterization of the CDs-Cyst-PAA^[30:70]^-M Conjugate and Precursors

The optical absorption and emission properties of CDs, CDs-Cyst, and CDs-Cyst-PAA^[30:70]^-M were evaluated on aqueous dispersions of each sample at a concentration of 0.1 mg mL^–1^. Absorption spectra were recorded in the range of 200–800 nm using a double-beam spectrophotometer (Shimadzu UV-2401PC). The 3-D emission spectra of CDs, CDs-Cyst and CDs-Cyst-PAA^[30:70]^-M, spanning from 350 to 850 nm, were acquired using a spectrofluorometer (Jasco FP-8500) under excitation wavelengths ranging from 340 to 600 nm with acquisition intervals of 10 nm. Fluorescence spectra of CDs-Cyst-PAA^[30:70]^-M (0.45 mg mL^–1^) were also acquired by varying the pH in the range of 3.0 to 9.0. This was accomplished by utilizing citrate buffer (10 mM), acetate buffer (10 mM), and phosphate buffer (10 mM) while adding NaCl as an ionic strength stabilizer (59 mM).

Similarly, fluorescence spectra of CDs-Cyst-PAA^[30:70]^-M were recorded by varying concentrations (10, 5, 1, 0.5, 0.1, 0.05 mg mL^–1^) in an artificial lysosomal fluid buffer at pH 5.0 (0.15 M NaOH, 0.055 M NaCl, 0.11 M citric acid, 8.74 × 10^–4^ M CaCl_2_, 2.1 × 10^–4^ M NaH_2_PO_4_ 7H_2_O, 2.74 × 10^–4^ M NaSO_4_, 1.1 × 10^–3^ M MgCl_2_, 6.4 × 10^–4^ M glycerol, 2.62 × 10^–4^ M sodium citrate dihydrate, 3.9 × 10^–4^ M sodium tartrate dihydrate, 7.58 × 10^–4^ M sodium lactate, 7.8 × 10^–4^ M sodium pyruvate).

### Assessment of the Complexing Ability of CDs-Cyst-PAA^[30:70]^-M for a siRNA Model

Complexation studies were conducted through a gel retardation assay using a 1.5% agarose gel supplemented with 0.1% ethidium bromide, as described above. Polyplexes were prepared by adding a dispersions of CDs-Cyst-PAA^[30:70]^-M at different concentrations (10 μL) to an equal volume of siRNA solution (100 mg mL^–1^). The CDs-Cyst-PAA^[30:70]^-M/siRNA weight ratios (*R*) investigated varied from 0 to 5. The preparation of CDs-Cyst-PAA^[30:70]^-M/siRNA complexes was carried out in 5% (w/v) glucose-containing 10 mM nuclease-free Hepes buffer, pH 7.4. Mixtures were gently pipetted to ensure thorough mixing. After 30 min of incubation at room temperature, 10 μL of each sample was placed into an agarose gel well and the electrophoretic run was performed under the same conditions described above. The electrophoretic runs were visualized using a UV transilluminator capturing micrographs using a digital camera. This analysis was repeated after a 24 h incubation at 37 °C.

### DLS and ζ-Potential Measurements

For the DLS measurements, polyplexes were prepared in 10 mM nuclease free Hepes buffer pH 7.4 with different polymer/siRNA (*R*) weight ratios, using a concentration of siRNA equal to 0.1 mg mL^–1^. The DLS measurement was performed on 50 μL of the sample at 25 °C with a Malvern Zetasizer NanoZS equipped with a 633 nm laser with a fixed scattering angle of 173°, using Dispersion Technology Software 7.02. Subsequently, for the ζ-potential, polyplexes were diluted with nuclease-free water up to 900 μL before performing ζ-potential measurements, recorded at 25 °C using the same apparatus as the DLS. The ζ-potential (mV) values were calculated from electrophoretic mobility using the Smoluchowski relation.

### Evaluation of the Redox Sensitivity under Reducing Conditions

The redox sensitivity of the CDs-Cyst-PAA^[30:70]^-M conjugate was evaluated after incubation of the CDs-Cyst-PAA^[30:70]^-M/siRNA polyplexes with 10 mM reduced glutathione (GSH) for 2 h. The samples (40 μL), prepared as described for the complexation study (either *R* = 3, *R* = 4, or *R* = 5) were mixed with 5 μL of 90 mM GSH and left to incubate at room temperature. At scheduled time intervals (0 and 2 h) 10 μL of the sample was withdrawn and analyzed by gel electrophoresis as described above for the complexing ability studies.

### Stability of the CDs-Cyst-PAA^[30:70]^-M/siRNA Polyplexes to the Polyanionic Exchange

Time-dependent stability of CDs-Cyst-PAA^[30:70]^-M/siRNA polyplexes to polyanionic exchange was assessed in the presence of albumin at a physiological concentration of 40 mg mL^–1^. Samples (40 μL), prepared in a manner consistent with the complexation study, utilizing various CDs-Cyst-PAA^[30:70]^-M//siRNA weight ratios (*R* 3, 4 and 5), were mixed with 5 μL of bovine albumin 200 mg mL^–1^. These mixtures were then allowed to incubate on an orbiting shaker at room temperature. At 0 and 2 h, 10 μL of the sample was retrieved and subjected to analysis through gel electrophoresis, as previously described.

### Cytocompatibility by MTS Assay

Cytocompatibility of the PAAs was evaluated in vitro on 16-HBE and MCF-7, while for the CDs-Cyst-PAA^[30:70]^-M and CDs-Cyst-PAA^[30:70]^-M/siRNA polyplexes, the cytocompatibility was evaluated on HeLa, 16-HBE, and MCF-7 cell lines. PAA^[50:50]^ and PAA^[30:70]^ samples were prepared at different concentrations (range 250–25 μg mL^–1^) in DMEM supplemented with 10% FBS and 2 mM l-glutamine. However, CDs-Cyst-PAA^[30:70]^-M samples (range 50–5 μg mL^–1^) were prepared in DMEM supplemented with 10% FBS and 2 mM l-glutamine. The CDs-Cyst-PAA^[30:70]^-M/siRNA samples were prepared by mixing siRNA solution (0.2 mg mL^–1^) in RNAase-free water with CDs-Cyst-PAA^[30:70]^-M in 20 mM Hepes at various concentrations to obtain different CDs-Cyst-PAA^[30:70]^-M/siRNA weight ratios (*R*3, *R*5, *R*7, *R*10, *R*15). Each sample was incubated for 30 min. Then, Opti-ME medium was added to have a final siRNA concentration of 200 nM.

Each cell line was seeded on a 96-well plates with a cell density of 1.5 × 10^4^ per well and cultured in DMEM at 37 °C and 5% CO_2_. After 24 h, the culture medium was replaced with samples (200 μL) and cells were incubated for additional 48 h. The dispersion was removed and each well was washed three times with DPBS, pH 7.4. Then, cell viability was evaluated by MTS assay, treating each well with 100 μL of fresh DMEM and 20 μL of MTS solution. After 2 h of incubation, the absorbance at 492 nm was measured by using a microplate reader (Multiskan, Thermo, U.K.), and the cell viability was calculated considering the absorbance of untreated cells (negative control) as 100% viability.

### Cell Uptake Study by Fluorescence Microscopy

The cellular uptake of the CDs-Cyst-PAA^[30:70]^-M conjugate was investigated using HeLa cells. Cells were seeded in an 8-well plate at a density of 5000 cells per well in DMEM supplemented with 10% FBS. After a 24 h incubation period, the culture medium was aspirated, and cells were subsequently exposed to 200 μL of CDs-Cyst-PAA^[30:70]^-M in DMEM containing 10% FBS and 2 mM l-glutamine at a concentration of 0.1 mg mL^–1^. After 2, 6, or 24 h, cells underwent three rounds of gentle washing with sterile DPBS and were fixed with 4% formaldehyde for 5 min. The formaldehyde solution was then aspirated and cells were again washed three times with DPBS. Cellular observations were performed using an Axio Vert.A1 fluorescence microscope (Zeiss), equipped with a 100× magnification objective. Micrographs were captured with an Axio Cam MRm (Zeiss) camera.

Similarly, the cellular uptake of the CDs-Cyst-PAA^[30:70]^-M/siRNA polyplexes, prepared as described in the cell viability assay with an *R*5 weight ratio, was also evaluated using the same procedure.

### Western Blot Analysis

HeLa cells were seeded in a 24-well plate at a density of 10,000 cells per well (0.800 mL per well) and incubated at 37 °C and 5% CO_2_. After 24 h, the culture medium in each well was replaced with an aqueous dispersion containing CDs-Cyst-PAA^[30:70]^-M//siRNA polyplexes (1.330 mL per well, *R* = 5, 200 nM siRNA). As a negative control, untreated cells were included. After this time, cells in each well were washed with DPBS at pH 7.4 three times, subjected to trypsinization, and subsequently harvested. Postharvesting, cells were washed with cold PBS at pH 7.4 and treated with cell lysis buffer (0.5% sodium deoxycholate, 150 mM NaCl, 0.1% SDS, 1% NP-40, 50 mM Tris–HCl, pH 7.4, 20 mM NaF, 50 mM glycerophosphate, 20 mM EGTA, 0.5 mM PMSF, 1 mM DTT and 1 mM sodium orthovanadate) containing phosphatase and protease inhibitors. Samples were collected, transferred to microtubes, and vigorously vortexed for 15 s at 10 min intervals, repeating this process three times. The samples were then centrifuged at 12,000 rpm, at 4 °C, for 30 min. The protein concentration in the supernatant was determined using the Bradford assay. Subsequently, 80 μg of protein samples was subjected to SDS–PAGE and transferred to a nitrocellulose membrane.

For the immunoblot, the membrane was incubated overnight at 4 °C in a blocking solution containing 5% skim milk. This was followed by a 1 h incubation with anti-Bcl-2 monoclonal antibody (diluted at 1/200) at room temperature. The blots were then washed twice with Tris/Tween 20-buffered saline (TBST) and incubated with a 1:2000 dilution of horseradish peroxidase (HRP)-conjugated anti-IgG antibody for 1 h at room temperature. After an additional five washes with TBST, the blots were developed using enhanced chemiluminescence (Amersham Life Science, Arlington Heights, IL, USA). Immunoreactions were also conducted using β-actin antibodies (clone C4, Cat no. SC-47778, Santa Cruz Biotechnology) as loading controls.^44^

### Statistical Data Analysis

All the experiments were repeated at least three times. All data are expressed as the means ± standard deviation. All data were analyzed by Student’s *t*-test using Microsoft Excel software *p* < 0.05(*), *p* < 0.01(**) and *p* < 0.001(***).

## Results and Discussion

### Synthesis and Characterization of CDs-Cyst

Gd-doped CDs of 5.1 nm diameter were obtained by the solvothermal decomposition of citric acid and urea in the presence of Gd(III) ions (Figure S1). According to previous reported studies, the incorporation of Gd^3+^ ions within the core lattice and the surface structure was studied combining FT-IR, ^1^H NMR, and HR-TEM analyses (Figure S2a–c), demonstrating that Gd^3+^ ions are prevailingly entrapped in the CDs core, resulting in a unique lattice structure induced by stable bonds with the doping agent, and that CDs possess COOH groups on the surface, useful for further conjugation with polymer chains.^29^ In particular, the typical vibration bands of asymmetric C=O stretching, attributable to carboxyl (1715 cm^–1^) and amide I (1665 cm^–1^) groups, OH and NH stretching (3400 and 3200 cm^–1^), C–N (1385 cm^–1^) stretching, and N–C–O (1200 cm^–1^) (Figure S2a) imply that the surface of CDs is rich in polar functional groups suitable for easier engineering of the surface, as they confer high dispersibility in different solvents and can be easily functionalized by orthogonal synthetic protocols. It has been demonstrated that these CDs, apart from the emission of a bright blue-to-green light with a quantum yield of 11.5% (Figure S2d,e), are endowed with a relaxation time *T*_1_ and the corresponding relaxation rate *R*_1_ (=1/*T*_1_) significantly improved (about 4-fold higher than the reference) if compared with a clinical MRI standard such as gadobutrol (11.3 ± 0.2 vs 3.3 ± 0.2 mM^–1^ s^–1^ in aqueous dispersion, respectively).^29^ Thus, the combination of MRI and FLI contrast exhibited by these peculiar CDs, together with the ultrasmall dimensions below the renal cutoff, potentially allows image-guided theranostic approaches for personalized and precise siRNA delivery.

**Figure 1:**
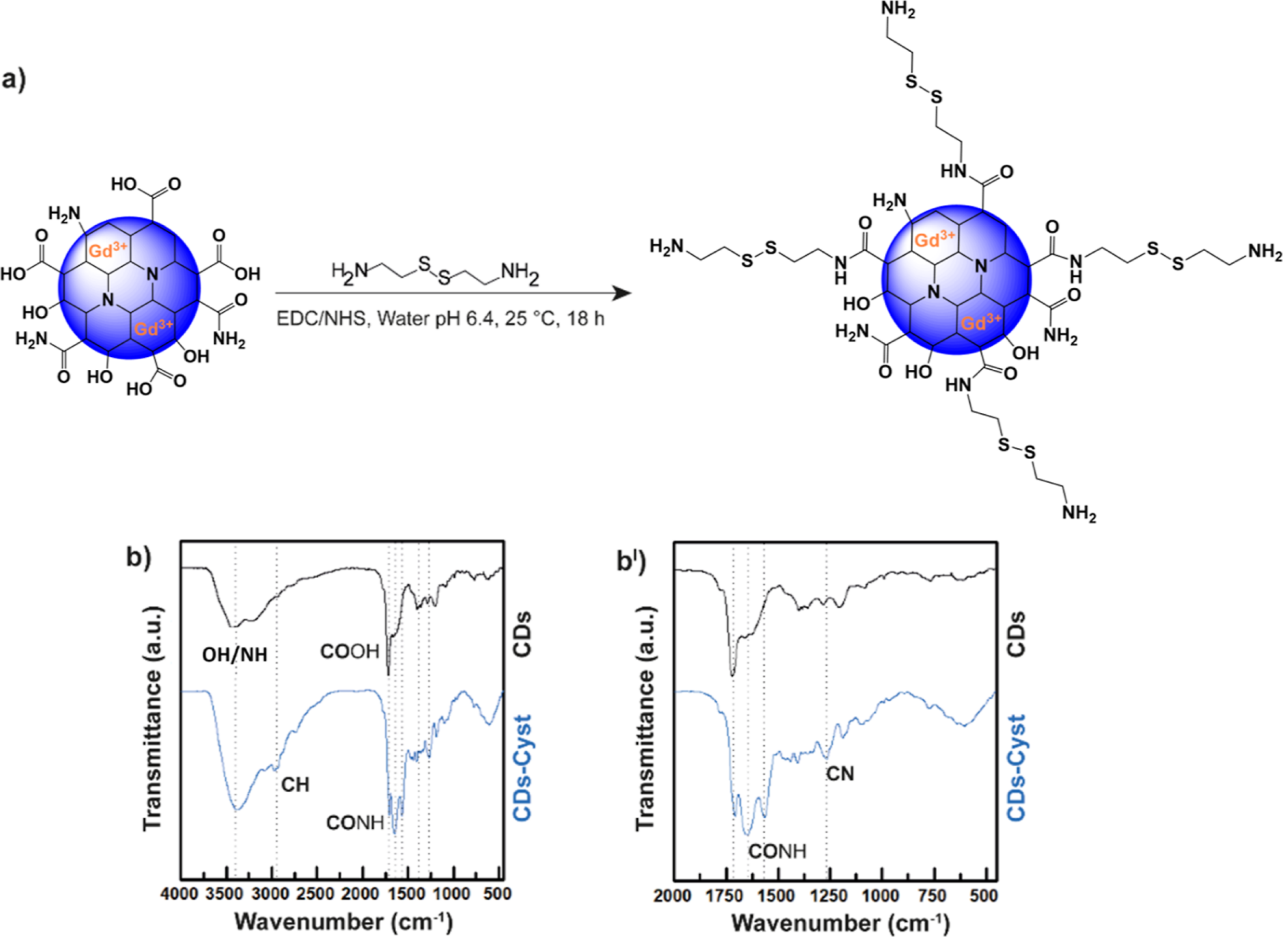
Scheme of synthesis for CDs-Cyst (a). FT-IR spectra of CDs and CDs-Cyst (b,b′).

CDs alone possess an anionic surface due to COO^–^ functions that does not permit the complexation of siRNAs, as the latter are also anionic. Hence, the surface of CDs was engineered with amphoteric PAAs, which are predominantly cationic and capable of reversibly binding siRNA under physiological conditions. CDs were first functionalized on the surface with cystamine pendants by amide coupling between the surface carboxyl groups of CDs and an amine group of cystamine, using EDC and NHS as coupling agents (Figure 1). Cystamine was chosen as a linker because it provides primary amine groups that are essential for the aza-Michael-type addition reaction with PAAs, facilitating the formation of stable and TME-responsive CDs-PAA amphoteric conjugates. Indeed, S–S bonds serve as bioreducible linkages that, when exposed to the reducing agent glutathione (GSH) within tumors, undergo cleavage, thus triggering both the release of siRNA/PAA polyplexes (in turn, GSH sensitive) and facilitating the liberation of ultrasmall bioeliminable CDs. The effective surface functionalization was confirmed by FT-IR analysis. As reported in Figure 1b,b′, the nude CDs’ spectrum is characterized by several characteristic bands relative to carboxyl groups (OH stretching at 3400 cm^–1^ and CO stretching at 1750 cm^–1^) and amide groups (CO stretching at 1636 cm^–1^). The spectrum of CDs-Cyst displayed marked peaks related to the stretching of the amide bond (1636–1578 cm^–1^), accompanied by a reduction of the carboxylic hydroxyl stretching (3214 cm^–1^) and aliphatic C–H vibrations (2934 cm^–1^), confirming that the amide coupling with cystamine moieties successfully occurred. The NMR analysis supports the conjugation with cystamine, as the CD-Cys spectrum shows the typical peaks of cystamine methylenes at 3.4 and 3.0 ppm (Figure S3).

Moreover, the exclusive monoaddition of cystamine chains to individual dots, without engaging in dot-to-dot cross-linking reactions, was validated through AMF analysis. As illustrated in Figure S4, the average diameter of CDs-Cyst was about 6.1 ± 0.4 nm, slightly exceeding that of the initial CDs (5.1 nm).

### Synthesis and Characterization of PAAs

**Figure 2:**
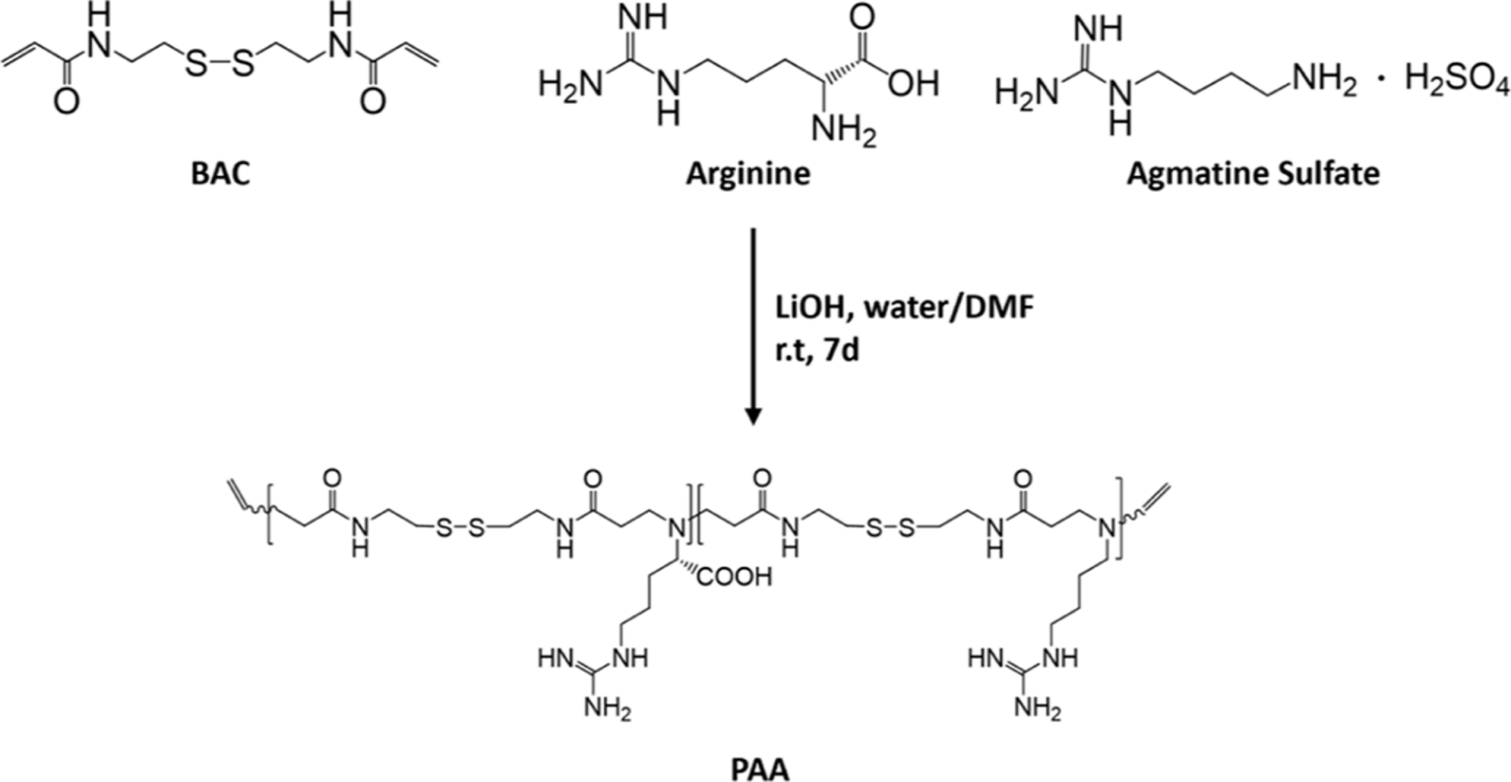
Synthetic pathway adopted for the synthesis of the PAA copolymers.

PAAs are a family of biocompatible and biodegradable synthetic polymers obtained by Michael-type polyaddition of primary or secondary amines with bis(acrylamide)s, where the appropriate choice of monomeric precursors allows us to obtain polymeric structures with tunable chemical, physical, and biological properties.^24^ Among these, amphoteric PAAs have been studied for their exceptional high biocompatibility if compared with the polycation counterpart and for their capability to efficiently and reversibly bind nucleic acids both in vitro and vivo.^25,45^ Besides, PAAs bearing disulfide bonds within their repeating units have been proposed in stimuli-sensitive drug delivery applications because they sharply degrade into short fragments when exposed to conditions mimicking the TME (i.e., 10 mM GSH).^28^ Thus, combining the effective complexing abilities in a unique PAA, the high cytocompatibility of amphoteric PAAs, and the rapid degradation of PAAs which contain disulfide brings would lead to bioreducible PAAs capable of preferential releasing siRNA in a TME-sensitive manner. With this in mind, in this work, we synthesized three distinct amphoteric, copolymeric, and linear PAA oligomers through the polyaddition of BAC with l-arginine and agmatine, using an increasing l-arginine/agmatine molar ratio (Figure 2). In particular, 30:70, 50:50, and 70:30 ratios were adopted so as to study the effect of the average charge balance on their cytocompatibility and siRNA complexing ability. Usually, the more cationic the polymer, the better it complexes, whereas cytotoxicity increases conversely.^22,23,25,46,47^ The synthetic stepwise polyaddition strategy adopted for the synthesis of the PAA^[70:30]^, PAA,^[50:50]^ and PAA^[30:70]^ oligomers involves the use of an excess of bis-acrylamides (BAC) to yield acrylamide-terminated chains consisting of approximately 5 repeating units.

The structure of the PAAs was attained by ^1^H- and ^13^C NMR in order to estimate the average number of repeating units per chain and to extrapolate percentages of arginine and agmatine compared to the pre-established composition. The ^1^H NMR (Figures S5 and S6) spectrum of PAA^[70:30]^, PAA,^[50:50]^ and PAA^[30:70]^ confirms the effective Michael addition between the terminal double bonds of BAC and the terminal primary amine of arginine and agmatine, as demonstrated by the peak at δ 3.45 relating to the methylenes involved in the C–N–C bond and the ratio between the peaks of the terminal residual double bonds and the peculiar peaks of repeating units. In detail, the effective functionalization in arginine (A) and agmatine (Ag) repeating units, calculated by comparing the integrals of the peak at either 3.75 ppm (1H, A) or 1.81 ppm (2H, Ag) with the integral of the peak at 2.76–3.0 ppm (4H, BAC), was in agreement with the theoretical stoichiometry adopted (Table 2.). Besides, the ratio of the areas of the peaks at δ 6.30–5.60/δ 1.88–1.56 were considered to extrapolate the number of repeating units and the corresponding average molecular weight of each PAA. As reported in Table 2, according to the ^1^H NMR data, each PAAs consists of 5 repeating units with an estimated number-averaged molecular weight of 2000–2100 and different percentages of arginine and agmatine in accordance with the established composition. The average molecular weight of the PAA oligomers was lower than that predicted by the Carothers relationship (eq 1) for step polymerization and 100% conversion (*p* = 1)
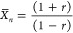
1where (*r*) is the critical composition ratio, defined as the molar ratio between amine and acrylamide functions.

**Table 2:** Number of Repeating Units, Arginine Repeating Units (A), Agmatine (Ag) Repeating Units, Molecular Weight, and Polydispersity Index of Each PAA Extrapolated by ^1^H NMR and SEC Analyses

samples	no repeating unit^a^	% A repeating units^a^	% Ag repeating units^a^	*M̅*_n_^a^	*M̅*_n_^b^	PD^b^
PAA^[70:30]^	5	68	32	2100	1200	1.19
PAA^[50:50]^	5	47	53	2000	1300	1.16
PAA^[30:70]^	5	26	74	2000	1300	1.19

a Obtained by ^1^H NMR analysis.

b Obtained by SEC analysis from a calibration curve of PEG standards.

In fact, all reactions were performed fixing *r* = 0.95, implying that the  of each oligomer should have been 39 (roughly 8-fold higher). Hence, for this particular reaction, the degree of polymerization was controlled empirically by inducing the precipitation of pentamers with number-averaged molecular weight of 2100 (*p* < 1) due to the chosen reaction conditions adopted (i.e., solvent mixture and concentration).

The average molecular weight distribution of each PAA was also confirmed by SEC (Table 2). SEC data align with the findings extrapolated from NMR analyses, affirming the efficacy of the synthetic approach in achieving PAAs with precise molecular weight control and reduced polydispersity.

### Selection of the PAA with the Highest Complexation Efficiency for a Model siRNA

From the acid–base standpoint, the arginine-bearing repeating units exhibit three ionizable groups with self-buffering properties: one carboxyl group, one tertiary amine group, and a guanidine group. However, the agmatine repeating units feature two ionizable cationic groups (a tertiary amine group and a guanidine group). Usually, the acid–base properties of the R side chain of this class of PAAs remain consistent regardless of the acrylamide used, accounting for experimental error.^48^ Given that PAAs with the same side chains have undergone extensive characterization regarding their acid–base properties, we can speculate that the arginine repeating units have p*K*_a1_ = 5.6, p*K*_a2_ = 6.9, and p*K*_a2_ > 12 (corresponding to the carboxyl group, tertiary amine group, and guanidine group, respectively).^23,49^ PAAs with agmatine repeating units exhibit p*K*_a1_ = 7.3 and p*K*_a2_ > 12 (Figure S7). Notably, both p*K*_a_ values of the tertiary amino groups and carboxylate groups fall within the endosomal acidification range (pH 7.4–5.1), imparting strong pH buffering properties. Consequently, PAAs typically demonstrate a buffering capacity in the range of 7.4–5.1 exceeding 20%, offering a distinct advantage over polyethylenimine (PEI), which exhibits a buffering capacity lower than 20%.^50^

To determine whether the synthesized PAAs can form electrostatic bonds with negatively charged siRNA strands, complexation studies were conducted using electrophoresis on agarose gel. To investigate this, polyplexes were formed by mixing equal volumes of two solutions: one containing a fixed siRNA concentration and the other with varying concentrations of each PAA, resulting in different polymer/siRNA weight ratios (*R*) ranging from 1.6 to 50. As shown in Figure S6, PAA^[70:30]^@siRNA polyplexes exhibited an electrophoretic profile similar to the control with free siRNA, with a slight reduction in migration speed observed only at the three highest ratios. In contrast, PAA^[50:50]^@siRNA displayed enhanced complexation capacity, as indicated by the reduced migration speed of siRNA at all tested ratios. PAA^[30:70]^@siRNA exhibited excellent complexation capacity, particularly at weight ratios from 6.25 to 50. It is noteworthy that the complexing ability improves as the percentage of agmatine increases (resulting in the absence of carboxyl/carboxylate groups in agmatine) and the positive charge balance increases. The remarkable complexing capabilities observed can be ascribed not solely to the inclusion of guanidine groups but also to the presence of disulfide bonds in the polymeric structure, in contrast to the methylene groups of methylenebis(acrylamide). The higher electronegativity of sulfur atoms, as compared to carbon, enables disulfide bonds to serve as hydrogen bond acceptors with nucleobases. Moreover, polymers containing disulfide bonds exhibit a higher degree of structural flexibility compared to their nonsulfur counterparts, which suggests that the polymer chains can adopt conformations favorable for interactions with nucleotide strands.^51^ Based on these findings, we opted to use PAA^[30:70]^ for the surface modification of CDs to enhance their siRNA complexation capability.

### Synthesis and Characterization of the Morpholine End-Capped CDs-PAA (CDs-Cyst-PAA^[30:70]^-M) Conjugate

**Figure 3:**
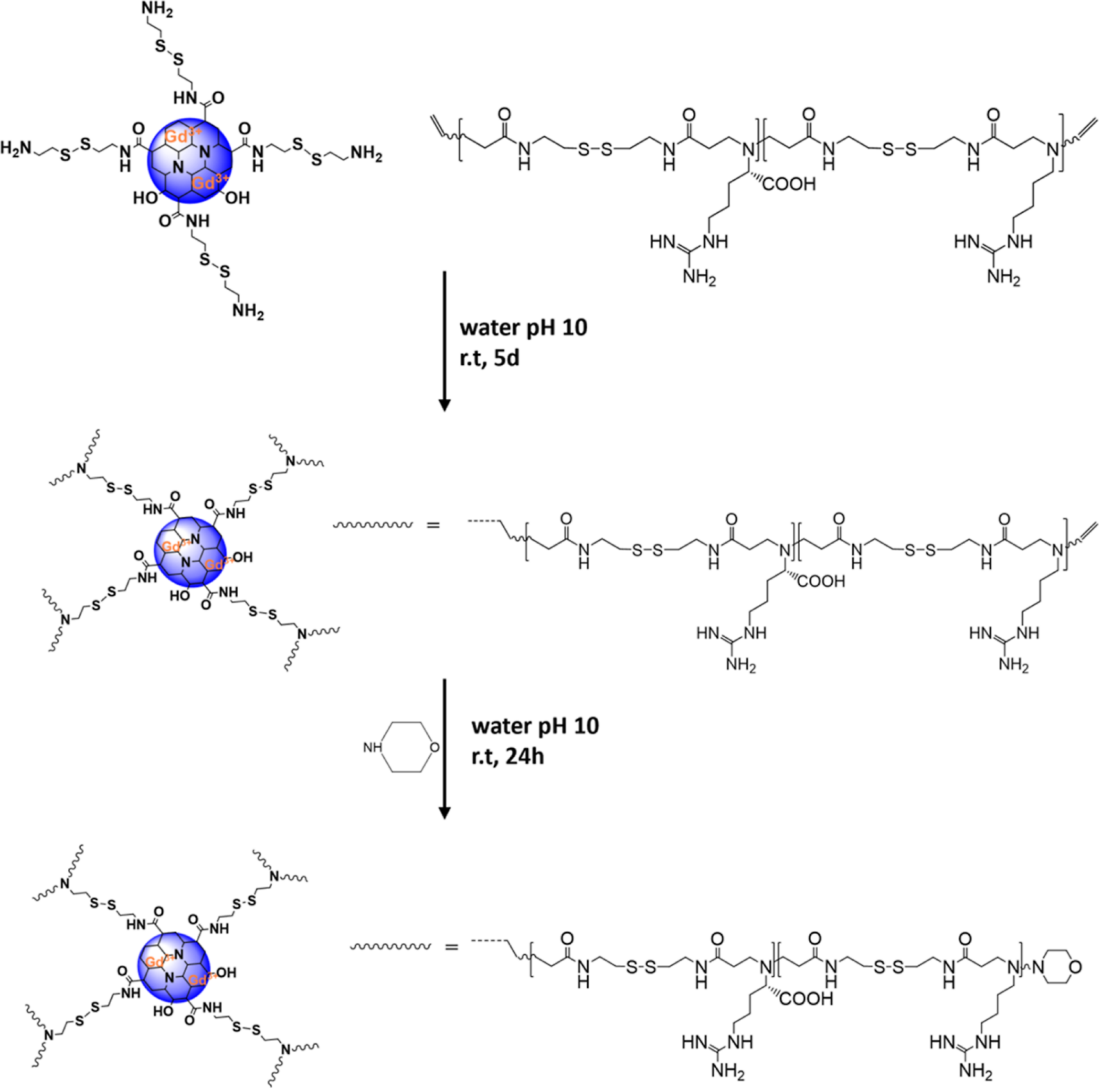
Scheme of synthesis for the CDs-Cyst-PAA^[30:70]^-M copolymers.

Since PAA^[30:70]^ showed the most favorable balance between complexation capacity and cytocompatibility profile (Figure S9), it was selected for the production of hybrid CDs-Cyst-PAA conjugates (CDs-Cyst-PAA^[30:70]^) (Figure 3).

As described above, CDs were functionalized with cystamine, chosen as a bioreducible and GSH-sensitive linker. The cystamine surface groups were exploited for the aza-Michael type addition to the acrylamide end-chains of the PAA^[30:70]^ oligomers via the primary amine group under mild basic conditions (pH 10.0). To prevent the cross-linking reactions between the two end-chains of PAA^[30:70]^ oligomers and amine groups of different CDs-Cyst, an excess of bauble bonds were employed. After that, the unbonded oligomers were removed by exhaustive dialysis. Using this synthetic strategy, each cystamine primary amine group would react with two oligomers yielding two-harm branched PAAs at the surface (Figure 3), thus eliciting efficient siRNA complexation at the CDs surface.

The effective conjugation of CDs-Cyst with PAA^[30:70]^ was confirmed by ^1^H NMR analysis (Figure S10). The successful addition of one double bond end-chain per PAA to the amine groups of the cystamine moieties on the CDs’ surface was confirmed by assessing the ratio of the peaks at δ 6.30–5.60/δ 1.88–1.56, which was reduced by half. The CDs-Cyst-PAA intermediate is of particular interest because the remaining double bond end-chains on the PAA pendants can be potentially utilized for subsequent conjugation with active targeting agents, such as aptamers, antibodies, and small bioactive molecules. However, the main aim of this work was to develop surface-engineered CDs with efficient image-guided siRNA complexation capability. Hence, we chose to deactivate the end-chains by capping them with a cationic and unreactive compound like morpholine. The saturation of the residual double bonds with morpholine was performed using an excess of morpholine, removed by dialysis, that was confirmed through ^1^H NMR by the disappearance of the resonances a and a′ (Figure S11).

The thermal stability of the final CDs-Cyst-PAA^[30:70]^-M conjugate was studied coupling differential scanning calorimetry coupled with thermogravimetric analysis (DSC-TGA) (Figure S12). Both the DSC and TGA thermograms show that, when subjected to heating, the conjugate was stable up to 230 °C, losing about 36% of the total mass within 240–500 °C. This weight loss is associated with an endothermic transformation, which can be attributed to the degradation of the polymer chains, thus indicating that PAAs-M oligomers are about 36% w/w of the final sample.

The amount of paramagnetic Gd^3+^ ions integrated in the CDs-Cyst-PAA^[30:70]^-M conjugate was evaluated spectrophotometrically, using XO tetrasodium salt as the metal indicator. In order to release the Gd^3+^ ions entrapped in the nanoparticle core, the sample of CDs-Cyst-PAA^[30:70]^-M was subjected to a mineralization process by dissolving the sample in 10% HNO_3_ under high-temperature treatment and then analyzed as per a report in the literature.^43^ By comparing the 573/433 nm absorbance ratio with a calibration curve generated using GdCl_3_ standards, it was calculated that Gd^3+^ makes up 0.23% w/w of CDs-Cyst-PAA^[30:70]^-M. This value is not far from previously reported gadolinium-doped CDs endowed with extraordinary MRI contrast properties (0.34–0.56%).^37,52^

### Optical Characterization of CDs-Cyst-PAA^[30:70]^-M Conjugates and Precursors

**Figure 4:**
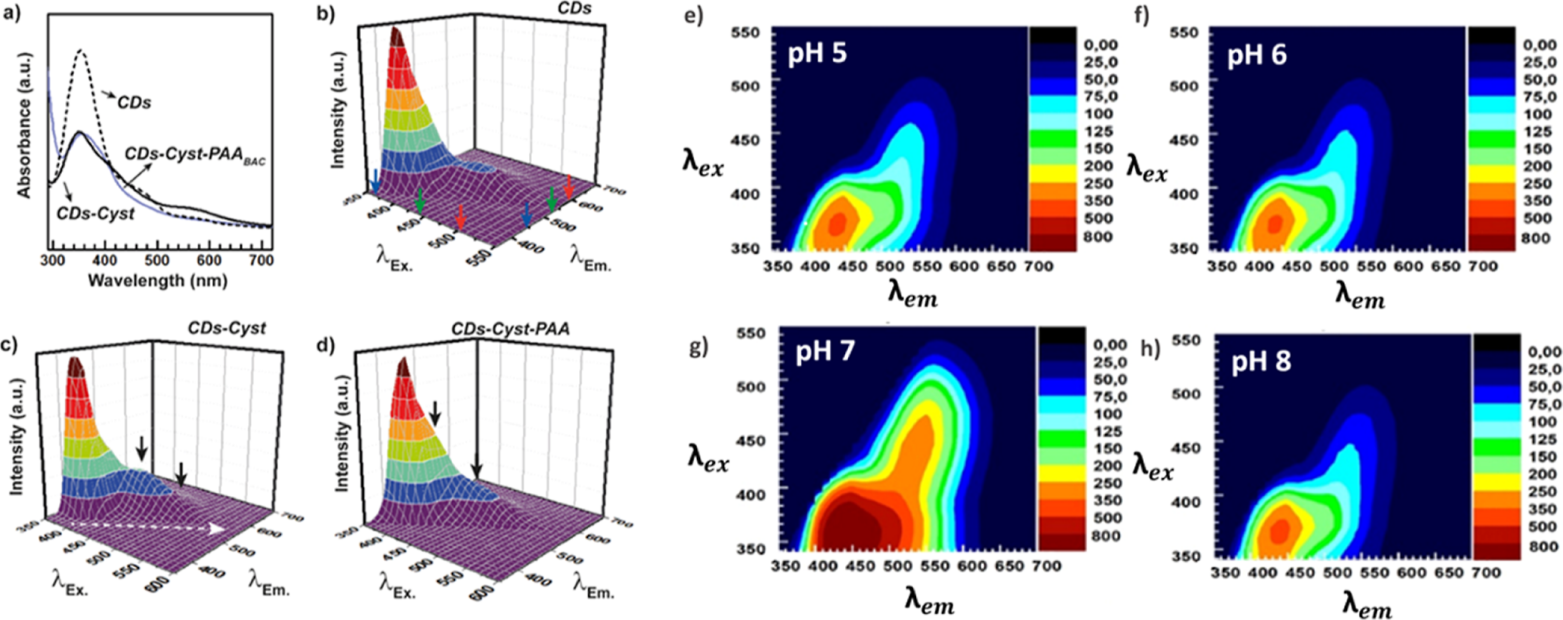
Optical characterization of CDs-Cys-PAA^[30:70]^-M and precursors: UV spectra (a) and 3-D emission spectra of CDs (b), CDs-Cyst (c) and CDs-Cyst-PAA^[30:70]^-M (d). 3D fluorescence emission spectra of CDs-Cyst-PAA^[30:70]^-M at different pH (e–g).

The virgin CDs showed a complex optical absorption spectrum that extends throughout the UV–vis range with peculiar absorption peaks at 350 nm and two bands at 460 and 540 nm. This multicolor emission feature allows fluorescence imaging, which is of particular interest in liquid biopsy and ex vivo diagnostic applications. After functionalization with cystamine (CDs-Cyst), the absorption spectrum is preserved, but with an increase of the blue absorption band at about 350 nm. On the contrary, for the CDs-Cyst-PAA^[30:70]^-M conjugate, an absorption profile similar to the nude CDs was observed. In particular, as showed in Figure 4, CDs showed a multicolor fluorescence profile from blue to red, with a particularly intense emission peak in the blue (450 nm) exciting at 350 nm and a less intense at 580 nm exciting beyond 500 nm. CDs-Cyst showed a red shift of the emission profile up to 610 nm as evidenced by comparing the ratio of the green emission band at 450 and the orange emission band at 500 nm to the blue emission peak at 350 nm. The 3-D fluorescence profile of the CDs-Cyst-PAA^[30:70]^-M conjugate showed an emission profile similar to CDs-Cyst, characterized by an increase of the fluorescence emission in orange and red regions in comparison with nude CDs.

The emission properties of CDs are usually influenced by the surface states of confined electrons, and thus changes measures in the emission spectra are directly correlated with the conjugation processes occurred on the CDs surface, as they involve surface functional groups.^53^ Besides, it is possible to hypothesize that these electronic transitions involve a coupling between the crystalline core and the surface groups.^54^ Therefore, the conjugation of CDs with PAA is not only functional for the purpose of gene delivery but also allows one to exploit the diagnostic properties of fluorescence imaging in vivo, given the shift of the emission peak to the of biological transparency window.

As the emission profile of CDs is based on quantum confinement effects that can be influenced by ionizable surface groups such as amphoteric PAAs, we proceeded to investigate how the emission bands depend on the pH of the medium and the consequent ionization of surface-conjugated PAAs. Fluorescence profiles were investigated as a function of pH, ranging from 5 to 8. As can be seen in Figure 4e–h, the conjugation with PAA implies an increase in fluorescence intensity at pH 7 throughout the emission range considered, with a red shift of the emission profile up to 640 nm by exciting at 500 nm. The pH-sensitive fluorescence observed for the CDs-Cyst-PAA^[30:70]^-M conjugate within various body pH conditions (e.g., 5.5 and 7.4 in TME and extracellular fluids, respectively) is a peculiar characteristic useful for biosensing applications. Certainly, this characteristic can also be synergized with the inherent pH-sensitive MRI contrast properties of the gadolinium-doped CDs, making it well-suited for in vivo theranostic applications.^29^

### Characterization of CDs-Cyst-PAA^[30:70]^-M/siRNA Complexes

To assess the complexation efficiency of CDs-Cyst-PAA^[30:70]^-M, agarose gel electrophoresis analysis was conducted. Equal volumes of two dispersions were mixed, one containing anti-Bcl2 siRNA at a fixed concentration and the other containing an increasing concentration of CDs-Cyst-PAA^[30:70]^-M, in order to obtain different polymer/siRNA weight ratios (*R*) within the range 1–5.

**Figure 5:**
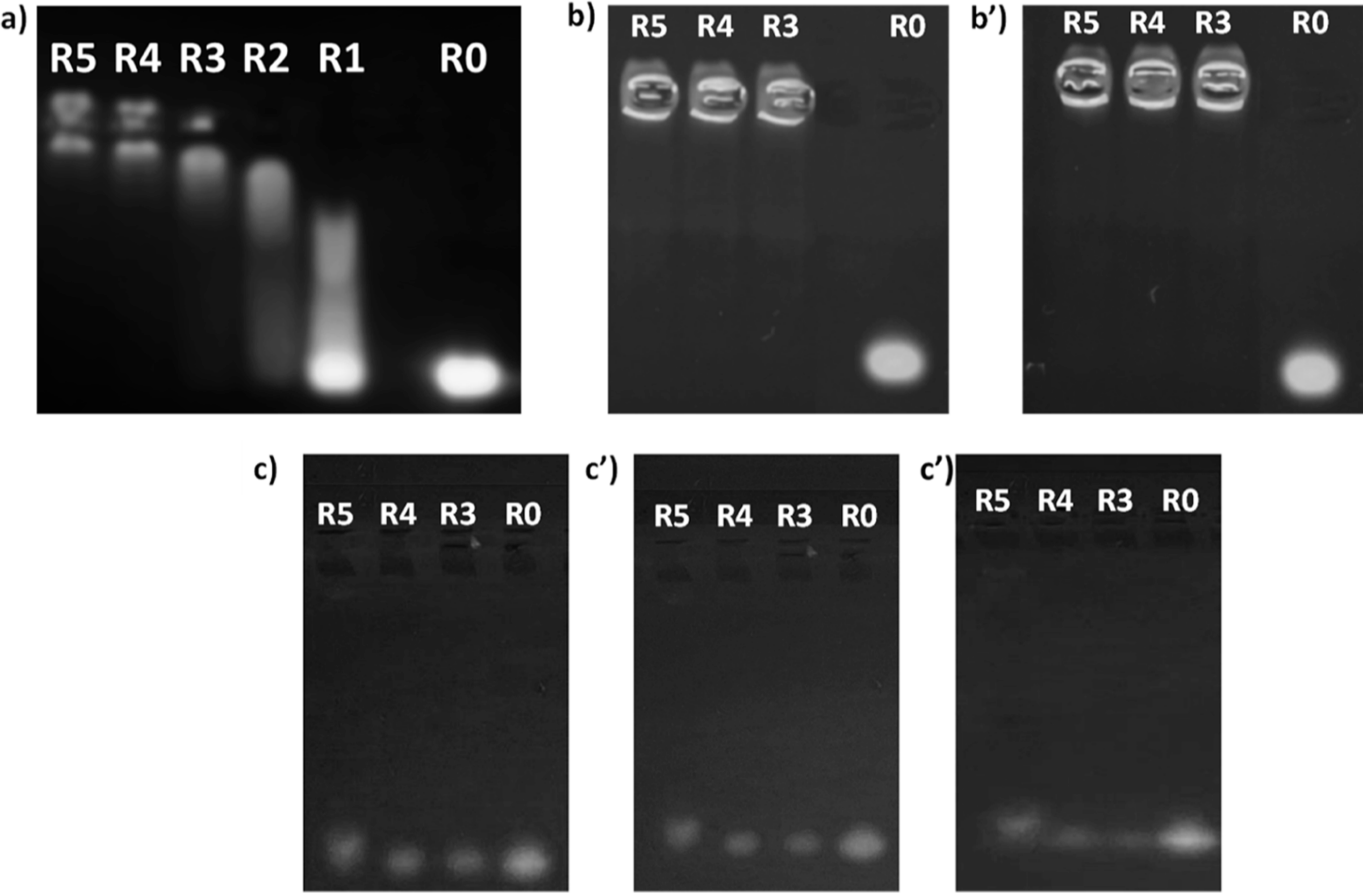
Gel retard assays: at different CDs-Cyst-PAA^[30:70]^-M/siRNA weight ratios (a); in the presence of albumin at time zero (b) and after 2 h (b′); in the presence of glutathione at time zero (c), after 2 h (c′) and after 6 h (c″). Free siRNA (*R*0) was used as the control.

As it can be seen in Figure 5, the CDs-Cyst-PAA^[30:70]^-M conjugate was able to strongly bind the siRNA strands starting from an *R* equal to 3, a ratio quite low if compared with other synthetic copolymers proposed as nonviral vectors for siRNA delivery.^55^ Specifically, taking into account that tertiary amines contribute to only 431.6%, and considering that for each repeat unit containing arginine, the positive charge of the guanidine group is counterbalanced by the negative charge of the carboxylate, this weight ratio corresponds to an N/P ratio of 1.9.^56^ The conjugation of PAAs with CDs positively influences the electrostatic interactions with siRNAs since the conjugated systems generate a retention of the electrophoretic migration of the nucleotide material much higher than that observed for the free PAAs (Figure S8). This is likely due to the cooperative binding of multiple PAA chains to siRNA oligomers on the CDs’ surface.

Given that the most common delivery route of siRNA-based therapies is the intravenous administration,^57^ it becomes necessary to understand what is the fate of the nanosystem as it comes into contact with elements in the bloodstream, such as plasma proteins. In particular, albumin has a polyanionic structure that could cause polyanionic exchange with siRNA. For this reason, the stability of CDs-Cyst-PAA^[30:70]^-M/siRNA polyplexes was established by incubating them with human albumin as a function of time. As depicted in Figure 5b,b′, following gel electrophoresis, no migration bands are discernible at any of the examined weight ratios, either at time zero or after 2 h of incubation. This confirms that the nanosystem remains stable in the presence of albumin, preventing the premature release of siRNA into the bloodstream.

With the aim of understanding whether the presence of glutathione, at a concentration typical of TME, could trigger the release of siRNA through the cleavage of disulfide bonds along the polymeric backbone, a time-dependent stability study was conducted. This study involved incubating CDs-Cyst-PAA^[30:70]^-M/siRNA nanosystems (prepared with *R*3, *R*4, and *R*5) with glutathione up to 6 h. As depicted in Figure 5c–c″, gel electrophoresis reveals that the release of siRNA is already evident at time zero. This behavior is observed even with longer incubation times, indicating that there are no reoxidation reactions that lead to the reorganization of the toroidal structures. The successful development of a redox-sensitive mechanism for siRNA release holds great promise as a strategy for the targeted delivery and controlled release of anticancer siRNA within the TME. This approach has the potential to trigger intracellular gene silencing of genes associated with tumor progression, offering a hopeful avenue for cancer therapy.

The average diameter and the surface charge of the CDs-Cyst-PAA^[30:70]^-M/siRNA polyplexes, obtained with different *R*, were evaluated by dynamic light scattering and ζ-potential measurements. As reported in Table 3, all polyplexes have an average hydrodynamic diameter of roughly 300 nm and an almost neutral ζ-potential in water.

**Table 3:** *Z*-Average, PDI, *Z* Potential of CDs-Cyst-PAA^[30:70]^-M/siRNA Polyplexes

CDs-Cyst-PAA^[30:70]^-M/siRNA weight ratio	*Z*-average (nm)	PDI	zeta potential (mV)
*R*5	391 ± 14	0.36 ± 0.03	–2.2 ± 6.2
*R*4	311 ± 6	0.24 ± 0.07	–1.2 ± 6.6
*R*3	331 ± 10	0.23 ± 0.05	3.3 ± 6.5

**Figure 6:**
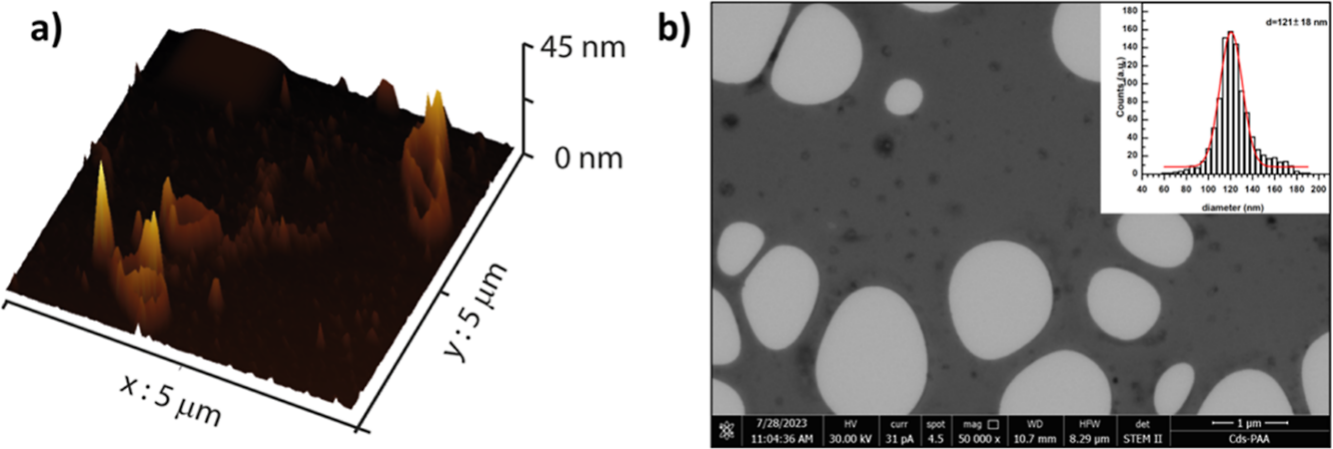
AFM (a) and STEM (b) of CDs-Cyst-PAA^[30:70]^-M/siRNA. The size distribution is reported in the inset (b).

The hydrodynamic diameter observed is usually associated with a suitable cell penetrating capability. Nonetheless, the morphological study of the CDs-Cyst-PAA^[30:70]^-M/siRNA, carried out by combining AFM and scanning transmission electron microscopy (STEM) analyses (Figure 6a,b), clearly reveals that the conjugate induces the formation of toroidal structures characterized by an anisotropic spatial distribution. In particular, CDs-Cyst-PAA^[30:70]^-M/siRNA toroids possess a longitudinal diameter of about 120 nm and average heights of 40 nm in the dried state.

### Uptake Study

**Figure 7:**
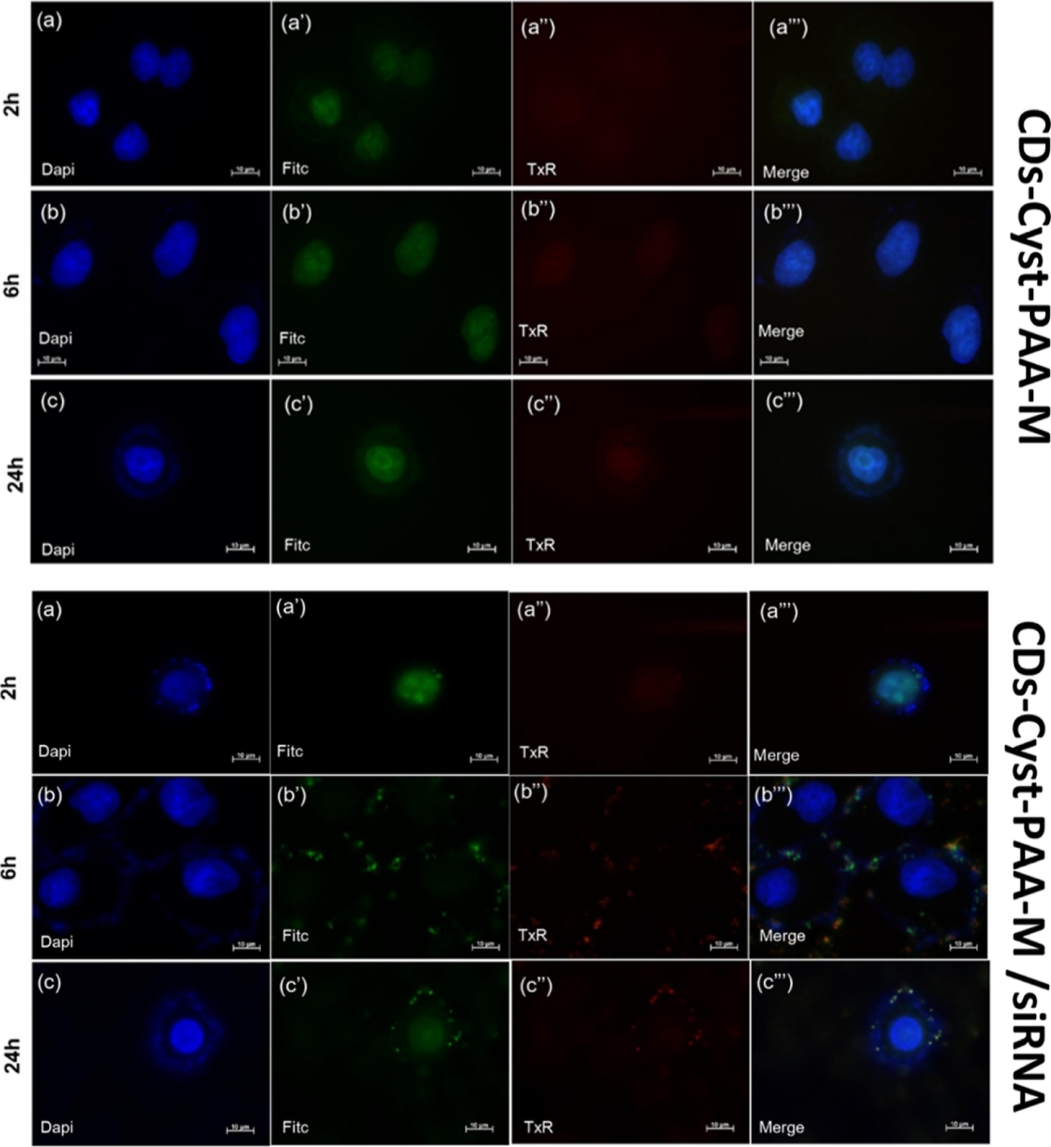
Cellular uptake of CDs-Cyst-PAA-M and CDs-Cyst-PAA-M/siRNA on HeLa after 2 (a–a‴), 6 (b,b″,b″), and 24 h (c,c′,c″,c″). The fluorescence in blue (a,b,c), green (a′,b′,c′), and red (a″,b″,c″) channels is due to CDs’ self-fluorescence.

The cellular internalization of both CDs-Cyst-PAA-M and the CDs-Cyst-PAA-M/siRNA interpolyelectrolyte complex was evaluated by fluorescence microscopy on the HeLa cell line, exploiting the multicolor fluorescence properties of CDs, which show a pronounced blue-to-red emission (Figure 4). After only 2 h of incubation (Figure 7a,a′,a″,a″), CDs-Cyst-PAA-M are efficiently internalized by cells, and more specifically inside nuclei, as evidenced by the blue and green fluorescence observed. After 6 h (Figure 7b,b′,b″,b″) the fluorescence profile is comparable to that observed at 2 h, while after 24 h (Figure 7c,c″,c″), the localization is also observed in peripheral areas. Regarding the CDs-Cyst-PAA-M/siRNA polyplexes, they reach cell nuclei, cytosol and are also confined within vesicular structures just 2 h after incubation (Figure 7a,a′,a″,a″). This is more evident both after 6 h (Figure 7b,b′,b″,b″) and 24 h (Figure 7c,c″,c″) of incubation, where an enhanced blue, green, and red fluorescence is observed. This might be ascribed to the accumulation of polyplexes inside intracellular organelles such as lysosomes, where the emission in the green–red region is visible only at a high concentration owing to the pH-dependent trend observed (Figures 4e–g and S13).

It is noteworthy that direct conclusions about the intracellular trafficking cannot be drawn from the seemingly distinct trends of the three fluorescence intensities (blue, green, and red) within the cells. This is due to the significantly different quantum yields of the three emission bands and because fluorescence is also influenced by the pH of various intracellular compartments (Figure 4e–h).

### Assessment of In Vitro Cytocompatibility and Bcl-2 Gene Silencing

**Figure 8:**
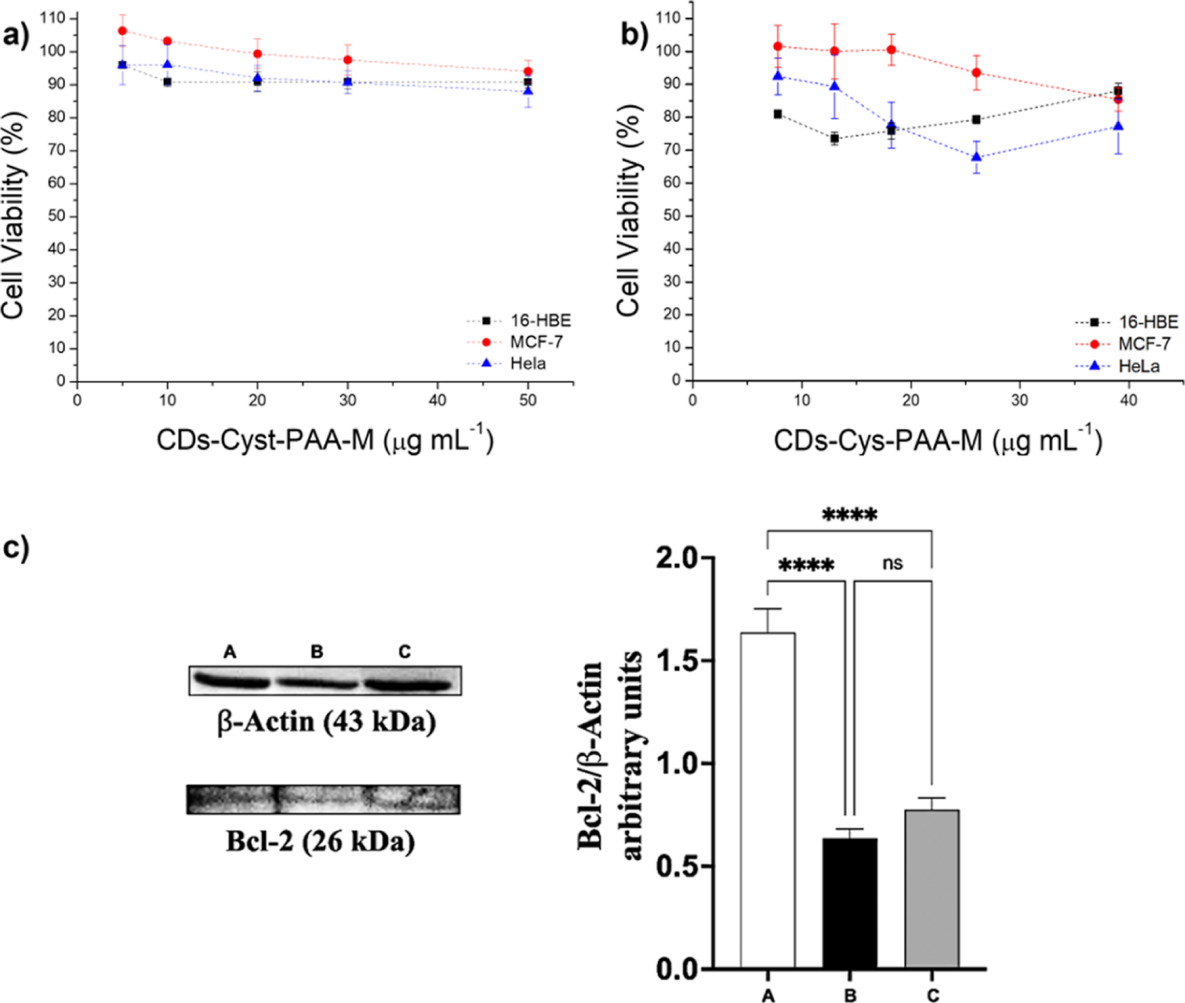
Cytocompatibility study CDs-Cyst-PAA^[30:70]^-M (a) and CDs-Cyst-PAA^[30:70]^-M/siRNA complexes (b) on 16-HBE, MCF-7, and HeLa cells after 48 h of incubation. (c) Representative image of Western blot analysis of Bcl-2 levels and Bcl-2 protein gene expression in HeLa cells: untreated cells (A), treated with siRNA (B), and with CDs-Cyst-PAA-M/siRNA (C); β-actin was used as an the internal control.

The in vitro cytocompatibility of the CDs-Cyst-PAA^[30:70]^-M and CDs-Cyst-PAA^[30:70]^-M/siRNA complexes was established on two different cancer cell lines, namely, HeLa and MCF-7, as well as a precancerous cell line (16-HBE). This assessment was conducted after 48 h of incubation (Figure 8).

The CDs-Cys-PAA^[30:70]^-M conjugate exhibits outstanding cytocompatibility for both the precancerous and cancer cell lines examined, as cell viability remained higher than 80% at all the tested concentrations. This indicates that the selected PAA for CDs’ functionalization strikes a favorable balance between delivery efficiency and in vitro toxicity by optimizing surface charge (Figure S8). Indeed, increasing the amount of agmatine in the copolymer results in an elevated positive charge (Figure S7), thereby enhancing siRNA complexation capability (Figure S8). Remarkably, despite the heightened cationic features of the PAAs^30:70^ copolymer compared to the PAAs^50:50^ and PAAs^70:30^, it maintains a favorable charge balance, ensuring high cytocompatibility (Figures 8 and S9). Besides, the cell viability of the CDs-Cyst-PAA^[30:70]^-M/siRNA interpolyelectrolyte complex, obtained with weight ratios from *R*3 to *R*15 and corresponding to a siRNA concentration of 200 nM, was also evaluated. As shown in Figure 8b, not surprisingly, in all tested cell lines, the cytocompatibility of the interpolyelectrolyte complex is maintained above the cytocompatibility threshold (70%). This is due to the low therapeutic efficacy reported by the sole treatment with anti-Bcl-2 siRNA. Indeed, inhibiting Bcl-2 alone is insufficient to attain effective antitumor activity and induce apoptotic cell death, as the apoptosis pathway involves various other proteins, including Bcl-xL, Mcl-1, Bax, and BH3.^58,59^ It does enhance the sensitivity of cancer cells to standard therapies with small molecules such as doxorubicin. However, a discrete reduction in cell viability is noticed as the concentration of CDs-Cys-PAA-M increases. This outcome could be associated with enhanced endocytosis, which, in turn, results in improved in vitro gene silencing capability. To assess whether the systems, once internalized, can induce gene silencing, Bcl-2 protein content was quantified by Western blot analysis (Figure 8c). The experiment was set with the same condition of cell viability assay, using high concentrations of siRNA as positive control. The high concentration of free siRNA in the positive control is intended to ensure internalization, while maintaining comparable concentrations between free siRNA and siRNA within the CD/siRNA complex during gene silencing experiments. Compared to untreated cells, the treatment with CDs-Cyst-PAA^[30:70]^-M/siRNA polyplexes reduced the expression of the Bcl-2 protein by half. This reduction is also observed in cells treated with free siRNA, thus suggesting that delivery with the synthesized vector does not alter its inherent silencing properties.

By combining the MRI/FL contrast properties of CDs-Cyst-PAA^[30:70]^-M/siRNA polyplexes, their high stability under conditions that mimic systemic administration with targeted and stimuli-responsive release, as well as the ability to silence genes through the release of siRNAs, it is conceivable that the proposed system has excellent potential as a theranostic platform for precision cancer therapy.

## Conclusions

This study is focused on the development of a hybrid nanosystem, consisting of CDs and amphoteric PAA oligomers, designed to serve as a stimuli-sensitive carrier for siRNA and as a theranostic agent in the image-guided treatment of tumors. The core of this nanosystem comprises ultrasmall gadolinium-doped CDs (5 nm) with MRI and fluorescence contrast properties, which were subsequently surface-functionalized with biodegradable GSH-sensitive amphoteric PAAs using cystamine as linkers. To achieve this, three distinct PAAs terminated with acrylamide functional groups were first synthesized by Michael-type polyaddition of BAC with arginine and agmatine, each with varying stoichiometric ratios of arginine and agmatine monomers, resulting in PAAs with different overall charges and siRNA complexation ability. To enhance polymer degradation in the reducing environments typical of cancer cells, BAC was chosen as the monomeric disulfide-containing building block susceptible to reduction in the presence of GSH. The structural and molecular weight distribution of the polymers was studied combining complementary techniques such as ^1^H NMR, ^13^C NMR, and SEC, validating the efficacy of the chosen synthetic approach.

The conjugation of CDs with the PAA oligomers with most efficient complexation ability was executed through a well-suited aza-Michael addition, using cystamine as linker. This yielded a monoaddition between one double bond of the PAA end-chains and the primary amine group of cystamine directly linked to the CDs’ surface by amide coupling. Finally, the residual double bonds were successfully deactivated with morpholine, as an additional cationic end-chain. This strategy avoided the dot-to-dot cross-linking reaction, yielding ordered core–shell nanosystems of a few nanometers. Evaluation of the optical properties revealed that surface passivation of CDs with both cystamine and PAA oligomers not only maintained but also enhanced the fluorescence properties. This enhancement was instrumental in extending the applicability of the nanosystems in fluorescence imaging by inducing a red shift in the fluorescence profile. Electrophoresis studies demonstrated the nanosystem’s capacity for complexation forming CDs-Cyst-PAA^[30:70]^-M/siRNA toroids of 120 nm. However, stability investigations in the presence of glutathione confirmed its ability to release siRNA in reducing environments, akin to tumor environments, by breaking the BAC and cystamine disulfide bridges within the polymer structure. Additionally, stability studies in the presence of albumin supported the notion that the nanosystem is resistant to premature siRNA release in the bloodstream, facilitating systemic administration.

Finally, through experiments conducted on three different cell lines, we demonstrated that CDs-Cyst-PAA^[30:70]^-M/siRNA can penetrate cancer cells with negligible cytotoxic effects and induce Bcl-2 silencing in vitro. In light of these promising results, the CDs-Cyst-PAA-M derivative emerges as a compelling candidate for a nonviral vector, seamlessly combining the diagnostic capabilities of CDs with the therapeutic potential of siRNA within a single platform.

## Supporting Information Available

Schematic synthetic route for the synthesis of CDs, characterization of CDs, ^1^H NMR of CDs-Cys, ^1^H NMR of PAA, ^13^C NMR of PAA, electrophoretic runs at different PAA/siRNA weight ratios, ^1^H NMR of CDs-Cyst-PAA^[30:70]^-M, DSC-TGA of CDs-Cyst-PAA^[30:70]^-M, and cytocompatibility study of PAA^[50:50]^ and PAA^[30:70]^, emission spectra of CDs-Cyst-PAA^[30:70]^-M in an artificial lysosomal fluid (PDF)

## References

[ref1] Jain D.; Prajapati S. K.; Jain A.; Singhal R. Nano-Formulated SiRNA-Based Therapeutic Approaches for Cancer Therapy. NanoTrends 2023, 1, 100006 10.1016/j.nwnano.2023.100006.

[ref2] Paul A.; Muralidharan A.; Biswas A.; Kamath B. V.; Joseph A.; Alex A. T. SiRNA Therapeutics and Its Challenges: Recent Advances in Effective Delivery for Cancer Therapy. OpenNano 2022, 7, 100063 10.1016/j.onano.2022.100063.

[ref3] Kara G.; Calin G. A.; Ozpolat B. RNAi-Based Therapeutics and Tumor Targeted Delivery in Cancer. Adv. Drug Delivery Rev. 2022, 182, 114113 10.1016/j.addr.2022.114113. 35063535

[ref4] Tekedereli I.; Alpay S. N.; Akar U.; Yuca E.; Ayugo-Rodriguez C.; Han H. D.; Sood A. K.; Lopez-Berestein G.; Ozpolat B. Therapeutic Silencing of Bcl-2 by Systemically Administered SiRNA Nanotherapeutics Inhibits Tumor Growth by Autophagy and Apoptosis and Enhances the Efficacy of Chemotherapy in Orthotopic Xenograft Models of ER (−) and ER (+) Breast Cancer. Mol. Ther.—Nucleic Acids 2013, 2, e121 10.1038/mtna.2013.45. 24022053 PMC4028016

[ref5] Pecot C. V.; Wu S. Y.; Bellister S.; Filant J.; Rupaimoole R.; Hisamatsu T.; Bhattacharya R.; Maharaj A.; Azam S.; Rodriguez-Aguayo C.; Nagaraja A. S.; Morelli M. P.; Gharpure K. M.; Waugh T. A.; Gonzalez-Villasana V.; Zand B.; Dalton H. J.; Kopetz S.; Lopez-Berestein G.; Ellis L. M.; Sood A. K. Therapeutic Silencing of KRAS Using Systemically Delivered SiRNAs. Mol. Cancer Ther. 2014, 13 (12), 2876–2885. 10.1158/1535-7163.MCT-14-0074. 25281617 PMC4416486

[ref6] Reyes-González J. M.; Armaiz-Peña G. N.; Mangala L. S.; Valiyeva F.; Ivan C.; Pradeep S.; Echevarría-Vargas I. M.; Rivera-Reyes A.; Sood A. K.; Vivas-Mejía P. E. Targeting C-MYC in Platinum-Resistant Ovarian Cancer. Mol. Cancer Ther. 2015, 14 (10), 2260–2269. 10.1158/1535-7163.MCT-14-0801. 26227489 PMC4596776

[ref7] Wang J.; Lu Z.; Wientjes M. G.; Au J. L. S. Delivery of SiRNA Therapeutics: Barriers and Carriers. AAPS J. 2010, 12 (4), 492–503. 10.1208/s12248-010-9210-4. 20544328 PMC2977003

[ref8] Bumcrot D.; Manoharan M.; Koteliansky V.; Sah D. W. Y. RNAi Therapeutics: A Potential New Class of Pharmaceutical Drugs. Nat. Chem. Biol. 2006, 2 (12), 711–719. 10.1038/nchembio839. 17108989 PMC7097247

[ref9] Van De Water F. M.; Boerman O. C.; Wouterse A. C.; Peters J. G. P.; Russel F. G. M.; Masereeuw R. Intravenously Administered Short Interfering RNA Accumulates in the Kidney and Selectively Suppresses Gene Function in Renal Proximal Tubules. Drug Metab. Dispos. 2006, 34 (8), 1393–1397. 10.1124/dmd.106.009555. 16714375

[ref10] Bartlett D. W.; Davis M. E. Effect of SiRNA Nuclease Stability on the in Vitro and in Vivo Kinetics of SiRNA-Mediated Gene Silencing. Biotechnol. Bioeng. 2007, 97 (4), 909–921. 10.1002/bit.21285. 17154307

[ref11] Hu B.; Zhong L.; Weng Y.; Peng L.; Huang Y.; Zhao Y.; Liang X. J. Therapeutic SiRNA: State of the Art. Signal Transduction Targeted Ther. 2020, 5 (1), 101–125. 10.1038/s41392-020-0207-x. PMC7305320 32561705

[ref12] Pengnam S.; Plianwong S.; Yingyongnarongkul B.; Patrojanasophon P.; Opanasopit P. Delivery of Small Interfering RNAs by Nanovesicles for Cancer Therapy. Drug Metab. Pharmacokinet. 2022, 42, 100425 10.1016/j.dmpk.2021.100425. 34954489

[ref13] Thomas C. E.; Ehrhardt A.; Kay M. A. Progress and Problems with the Use of Viral Vectors for Gene Therapy. Nat. Rev. Genet. 2003, 4 (5), 346–358. 10.1038/nrg1066. 12728277

[ref14] Cooper B. M.; Putnam D. Polymers for SiRNA Delivery: A Critical Assessment of Current Technology Prospects for Clinical Application. ACS Biomater. Sci. Eng. 2016, 2, 1837–1850. 10.1021/acsbiomaterials.6b00363. 33440520

[ref15] Chen G.; Wang Y.; Ullah A.; Huai Y.; Xu Y. The Effects of Fluoroalkyl Chain Length and Density on SiRNA Delivery of Bioreducible Poly(Amido Amine)s. Eur. J. Pharm. Sci. 2020, 152, 105433 10.1016/j.ejps.2020.105433. 32590121

[ref16] Cavalli R.; Primo L.; Sessa R.; Chiaverina G.; di Blasio L.; Alongi J.; Manfredi A.; Ranucci E.; Ferruti P. The AGMA1 Polyamidoamine Mediates the Efficient Delivery of SiRNA. J. Drug Targeting 2017, 25 (9–10), 891–898. 10.1080/1061186X.2017.1363215. 28817973

[ref17] Martello F.; Piest M.; Engbersen J. F. J.; Ferruti P. Effects of Branched or Linear Architecture of Bioreducible Poly(Amido Amine)s on Their in Vitro Gene Delivery Properties. J. Controlled Release 2012, 164, 372 10.1016/j.jconrel.2012.07.029. 22846986

[ref18] Li D.; Ahmed M.; Khan A.; Xu L.; Walters A. A.; Ballesteros B.; Al-Jamal K. T. Tailoring the Architecture of Cationic Polymer Brush-Modified Carbon Nanotubes for Efficient SiRNA Delivery in Cancer Immunotherapy. ACS Appl. Mater. Interfaces 2021, 13, 30284–30294. 10.1021/acsami.1c02627. 34170101

[ref19] Mauro N.; Chiellini F.; Bartoli C.; Gazzarri M.; Laus M.; Antonioli D.; Griffiths P.; Manfredi A.; Ranucci E.; Ferruti P. RGD-Mimic Polyamidoamine-Montmorillonite Composites with Tunable Stiffness as Scaffolds for Bone Tissue-Engineering Applications. J. Tissue Eng. Regener. Med. 2017, 11 (7), 2164–2175. 10.1002/term.2115. 26948844

[ref20] Mauro N.; Ferruti P.; Ranucci E.; Manfredi A.; Berzi A.; Clerici M.; Cagno V.; Lembo D.; Palmioli A.; Sattin S. Linear Biocompatible Glyco-Polyamidoamines as Dual Action Mode Virus Infection Inhibitors with Potential as Broad-Spectrum Microbicides for Sexually Transmitted Diseases. Sci. Rep. 2016, 6, 33393 10.1038/srep33393. 27641362 PMC5027566

[ref21] Ferruti P.; Mauro N.; Manfredi A.; Ranucci E. Hetero-Difunctional Dimers as Building Blocks for the Synthesis of Poly(Amidoamine)s with Hetero-Difunctional Chain Terminals and Their Derivatives. J. Polym. Sci., Part A: Polym. Chem. 2012, 50 (23), 4947–4957. 10.1002/pola.26325.

[ref22] Manfredi A.; Mauro N.; Terenzi A.; Alongi J.; Lazzari F.; Ganazzoli F.; Raffaini G.; Ranucci E.; Ferruti P. Self-Ordering Secondary Structure of d- and l-Arginine-Derived Polyamidoamino Acids. ACS Macro Lett. 2017, 6, 987–991. 10.1021/acsmacrolett.7b00492. 35650880

[ref23] Ferruti P.; Mauro N.; Falciola L.; Pifferi V.; Bartoli C.; Gazzarri M.; Chiellini F.; Ranucci E. Amphoteric, Prevailingly Cationic L-Arginine Polymers of Poly(Amidoamino Acid) Structure: Synthesis, Acid/Base Properties and Preliminary Cytocompatibility and Cell-Permeating Characterizations. Macromol. Biosci. 2014, 14 (3), 390–400. 10.1002/mabi.201300387. 24821667

[ref24] Ferruti P. Poly(Amidoamine)s: Past, Present, and Perspectives. J. Polym. Sci., Part A: Polym. Chem. 2013, 51 (11), 2319–2353. 10.1002/pola.26632.

[ref25] Cavalli R.; Primo L.; Sessa R.; Chiaverina G.; di Blasio L.; Alongi J.; Manfredi A.; Ranucci E.; Ferruti P. The AGMA1 Polyamidoamine Mediates the Efficient Delivery of SiRNA. J. Drug Targeting 2017, 25 (9–10), 891–898. 10.1080/1061186X.2017.1363215. 28817973

[ref26] Ferruti P.; Franchini J.; Bencini M.; Ranucci E.; Zara G. P.; Serpe L.; Primo L.; Cavalli R. Prevailingly Cationic Agmatine-Based Amphoteric Polyamidoamine as a Nontoxic, Nonhemolytic, and “Stealthlike” DNA Complexing Agent and Transfection Promoter. Biomacromolecules 2007, 8 (5), 1498–1504. 10.1021/bm061126c. 17388564

[ref27] Li R.; Wei F.; Wu X.; Zhou P.; Chen Q.; Cen Y.; Xu G.; Cheng X.; Zhang A.; Hu Q. PEI Modified Orange Emissive Carbon Dots with Excitation-Independent Fluorescence Emission for Cellular Imaging and SiRNA Delivery. Carbon 2021, 177, 403–411. 10.1016/j.carbon.2021.02.069.

[ref28] Emilitri E.; Ranucci E.; Ferruti P. New Poly(Amidoamine)s Containing Disulfide Linkages in Their Main Chain. J. Polym. Sci., Part A: Polym. Chem. 2005, 43 (7), 1404–1416. 10.1002/pola.20599.

[ref29] Mauro N.; Cillari R.; Gagliardo C.; Utzeri M. A.; Marrale M.; Cavallaro G. Gadolinium-Doped Carbon Nanodots as Potential Anticancer Tools for Multimodal Image-Guided Photothermal Therapy and Tumor Monitoring. ACS Appl. Nano Mater. 2023, 6 (18), 17206–17217. 10.1021/acsanm.3c03583. 37772264 PMC10526686

[ref30] Soares P. I. P.; Romão J.; Matos R.; Silva J. C.; Borges J. P. Design and Engineering of Magneto-Responsive Devices for Cancer Theranostics: Nano to Macro Perspective. Prog. Mater. Sci. 2021, 116, 100742 10.1016/j.pmatsci.2020.100742.

[ref31] Shanbhag P. P.; Jog S. V.; Chogale M. M.; Gaikwad S. S. Theranostics for Cancer Therapy. Curr. Drug Delivery 2013, 10 (3), 357–362. 10.2174/1567201811310030013. 23286214

[ref32] Mishra V.; Patil A.; Thakur S.; Kesharwani P. Carbon Dots: Emerging Theranostic Nanoarchitectures. Drug Discovery Today 2018, 23, 1219–1232. 10.1016/j.drudis.2018.01.006. 29366761

[ref33] Mauro N.; Utzeri M. A.; Varvarà P.; Cavallaro G. Functionalization of Metal and Carbon Nanoparticles with Potential in Cancer Theranostics. Molecules 2021, 26 (11), 3085–3135. 10.3390/molecules26113085. 34064173 PMC8196792

[ref34] Kang Z.; Lee S. T. Carbon Dots: Advances in Nanocarbon Applications. Nanoscale 2019, 11 (41), 19214–19224. 10.1039/C9NR05647E. 31513215

[ref35] Ji D. K.; Reina G.; Liang H.; Zhang D.; Guo S.; Ballesteros B.; Ménard-Moyon C.; Li J.; Bianco A. Gadolinium-Incorporated Carbon Nanodots for T1-Weighted Magnetic Resonance Imaging. ACS Appl. Nano Mater. 2021, 4 (2), 1467–1477. 10.1021/acsanm.0c02993.

[ref36] He X.; Luo Q.; Zhang J.; Chen P.; Wang H. J.; Luo K.; Yu X. Q. Gadolinium-Doped Carbon Dots as Nano-Theranostic Agents for MR/FL Diagnosis and Gene Delivery. Nanoscale 2019, 11 (27), 12973–12982. 10.1039/C9NR03988K. 31263818

[ref37] Yu C.; Xuan T.; Chen Y.; Zhao Z.; Liu X.; Lian G.; Li H. Gadolinium-Doped Carbon Dots with High Quantum Yield as an Effective Fluorescence and Magnetic Resonance Bimodal Imaging Probe. J. Alloys Compd. 2016, 688, 611–619. 10.1016/j.jallcom.2016.07.226.

[ref38] Kim S.; Choi Y.; Park G.; Won C.; Park Y. J.; Lee Y.; Kim B. S.; Min D. H. Highly Efficient Gene Silencing and Bioimaging Based on Fluorescent Carbon Dots in Vitro and in Vivo. Nano Res. 2017, 10 (2), 503–519. 10.1007/s12274-016-1309-1.

[ref39] Scialabba C.; Sciortino A.; Messina F.; Buscarino G.; Cannas M.; Roscigno G.; Condorelli G.; Cavallaro G.; Giammona G.; Mauro N. Highly Homogeneous Biotinylated Carbon Nanodots: Red-Emitting Nanoheaters as Theranostic Agents toward Precision Cancer Medicine. ACS Appl. Mater. Interfaces 2019, 11, 19854–19866. 10.1021/acsami.9b04925. 31088077

[ref40] Mauro N.; Andrea Utzeri M.; Sciortino A.; Cannas M.; Messina F.; Cavallaro G.; Giammona G. Printable Thermo- and Photo-Stable Poly(D,L-Lactide)/Carbon Nanodots Nanocomposites via Heterophase Melt-Extrusion Transesterification. Chem. Eng. J. 2022, 443, 136525 10.1016/j.cej.2022.136525.

[ref41] Sciortino A.; Gazzetto M.; Buscarino G.; Popescu R.; Schneider R.; Giammona G.; Gerthsen D.; Rohwer E. J.; Mauro N.; Feurer T.; Cannizzo A.; Messina F. Disentangling Size Effects and Spectral Inhomogeneity in Carbon Nanodots by Ultrafast Dynamical Hole-Burning. Nanoscale 2018, 10 (32), 15317–15323. 10.1039/C8NR02953A. 30069566

[ref42] Zhang L.; Lu Z.; Zhao Q.; Huang J.; Shen H.; Zhang Z. Enhanced Chemotherapy Efficacy by Sequential Delivery of SiRNA and Anticancer Drugs Using PEI-Grafted Graphene Oxide. Small 2011, 7 (4), 460–464. 10.1002/smll.201001522. 21360803

[ref43] Barge A.; Cravotto G.; Gianolio E.; Fedeli F. How to Determine Free Gd and Free Ligand in Solution of Gd Chelates. A Technical Note. Contrast Media Mol. Imaging 2006, 1 (5), 184–188. 10.1002/cmmi.110. 17193695

[ref44] Restivo I.; Tesoriere L.; Frazzitta A.; Livrea M. A.; Attanzio A.; Allegra M. Anti-Proliferative Activity of A Hydrophilic Extract of Manna from Fraxinus Angustifolia Vahl through Mitochondrial Pathway-Mediated Apoptosis and Cell Cycle Arrest in Human Colon Cancer Cells. Molecules 2020, 25 (21), 5055 10.3390/MOLECULES25215055. 33143282 PMC7663425

[ref45] Griffiths P. C.; Mauro N.; Murphy D. M.; Carter E.; Richardson S. C. W.; Dyer P.; Ferruti P. Self-Assembled PAA-Based Nanoparticles as Potential Gene and Protein Delivery Systems. Macromol. Biosci. 2013, 13 (5), 641–649. 10.1002/mabi.201200462. 23512337

[ref46] Ferruti P.; Marchisio M. A.; Duncan R. Poly(Amido-Amine)s: Biomedical Applications. Macromol. Rapid Commun. 2002, 23, 332–355. 10.1002/1521-3927(20020401)23:5/6<332::aid-marc332>3.0.co;2-i.

[ref47] Pettit M. W.; Griffiths P.; Ferruti P.; Richardson S. C. W. Poly(Amidoamine) Polymers: Soluble Linear Amphiphilic Drug-Delivery Systems for Genes, Proteins and Oligonucleotides. Ther. Delivery 2011, 2 (7), 907–917. 10.4155/tde.11.55. 22833902

[ref48] Ranucci E.; Ferruti P.; Lattanzio E.; Manfredi A.; Rossi M.; Mussini P. R.; Chiellini F.; Bartoli C. Acid-Base Properties of Poly(Amidoamine)s. J. Polym. Sci., Part A: Polym. Chem. 2009, 47 (24), 6977–6991. 10.1002/pola.23737.

[ref49] Franchini J.; Ranucci E.; Ferruti P.; Rossi M.; Cavalli R. Synthesis, Physicochemical Properties, and Preliminary Biological Characterizations of a Novel Amphoteric Agmatine-Based Poly(Amidoamine) with RGD-like Repeating Units. Biomacromolecules 2006, 7 (4), 1215–1222. 10.1021/bm060054m. 16602741

[ref50] Patil S.; Lalani R.; Bhatt P.; Vhora I.; Patel V.; Patel H.; Misra A. Hydroxyethyl Substituted Linear Polyethylenimine for Safe and Efficient Delivery of SiRNA Therapeutics. RSC Adv. 2018, 8 (62), 35461–35473. 10.1039/C8RA06298F. 35547911 PMC9087824

[ref51] Lin C.; Engbersen J. F. J. The Role of the Disulfide Group in Disulfide-Based Polymeric Gene Carriers. Expert Opin. Drug Delivery 2009, 6 (4), 421–439. 10.1517/17425240902878010. 19382884

[ref52] Jiang Q.; Liu L.; Li Q.; Cao Y.; Chen D.; Du Q.; Yang X.; Huang D.; Pei R.; Chen X.; Huang G. NIR-Laser-Triggered Gadolinium-Doped Carbon Dots for Magnetic Resonance Imaging, Drug Delivery and Combined Photothermal Chemotherapy for Triple Negative Breast Cancer. J. Nanobiotechnol. 2021, 19 (1), 64 10.1186/s12951-021-00811-w. PMC7923633 33653352

[ref53] Sciortino A.; Marino E.; Dam B. V.; Schall P.; Cannas M.; Messina F. Solvatochromism Unravels the Emission Mechanism of Carbon Nanodots. J. Phys. Chem. Lett. 2016, 7 (17), 3419–3423. 10.1021/acs.jpclett.6b01590. 27525451

[ref54] Sciortino A.; Marino E.; Dam B. V.; Schall P.; Cannas M.; Messina F. Solvatochromism Unravels the Emission Mechanism of Carbon Nanodots. J. Phys. Chem. Lett. 2016, 7 (17), 3419–3423. 10.1021/acs.jpclett.6b01590. 27525451

[ref55] Cavallaro G.; Sardo C.; Craparo E. F.; Porsio B.; Giammona G. Polymeric Nanoparticles for SiRNA Delivery: Production and Applications. Int. J. Pharm. 2017, 525 (2), 313–333. 10.1016/j.ijpharm.2017.04.008. 28416401

[ref56] Ferruti P.; Mauro N.; Falciola L.; Pifferi V.; Bartoli C.; Gazzarri M.; Chiellini F.; Ranucci E. Amphoteric, Prevailingly Cationic L-Arginine Polymers of Poly(Amidoamino Acid) Structure: Synthesis, Acid/Base Properties and Preliminary Cytocompatibility and Cell-Permeating Characterizations. Macromol. Biosci. 2014, 14 (3), 390–400. 10.1002/mabi.201300387. 24821667

[ref57] Tatiparti K.; Sau S.; Kashaw S. K.; Iyer A. K. SiRNA Delivery Strategies: A Comprehensive Review of Recent Developments. Nanomaterials 2017, 7, 77 10.3390/nano7040077. 28379201 PMC5408169

[ref58] Hafezi S.; Rahmani M. Targeting BCL-2 in Cancer: Advances, Challenges, and Perspectives. Cancers 2021, 13, 1292–1315. 10.3390/cancers13061292. 33799470 PMC8001391

[ref59] Liu Q.; Osterlund E. J.; Chi X.; Pogmore J.; Leber B.; Andrews D. W. Bim Escapes Displacement by BH3- Mimetic Anti-Cancer Drugs by Double-Bolt Locking Both Bcl-XL and Bcl-2. Elife 2019, 8, 1–30. 10.7554/elife.37689. PMC6414199 30860026

