# Supplementary material for: Polyamidoamine-Carbon Nanodot Conjugates with Bioreducible Building Blocks: Smart Theranostic Platforms for Targeted siRNA Delivery
Source: Biomacromolecules. 2024 Jan 5;25(2):1191–204. doi: 10.1021/acs.biomac.3c01185 (PMC10865362; doi:10.1021/acs.biomac.3c01185)
Supplement: Supplementary file 1 — bm3c01185_si_001.pdf [file bm3c01185_si_001.pdf]

Supporting information

Polyamidoamine-Carbon Nanodots Conjugates with Bioreducible Building Blocks: Smart Theranostic Platforms for Targeted siRNA Delivery

Salvatore Emanuele Drago[1], Mara Andrea Utzeri[1], Nicolò Mauro[1]* and Gennara Cavallaro[1]

[1]Lab of Biocompatible Polymers, Department of Biological, Chemical and Pharmaceutical Sciences and Technologies (STEBICEF), University of Palermo, via Archirafi 32, 90123 Palermo, Italy

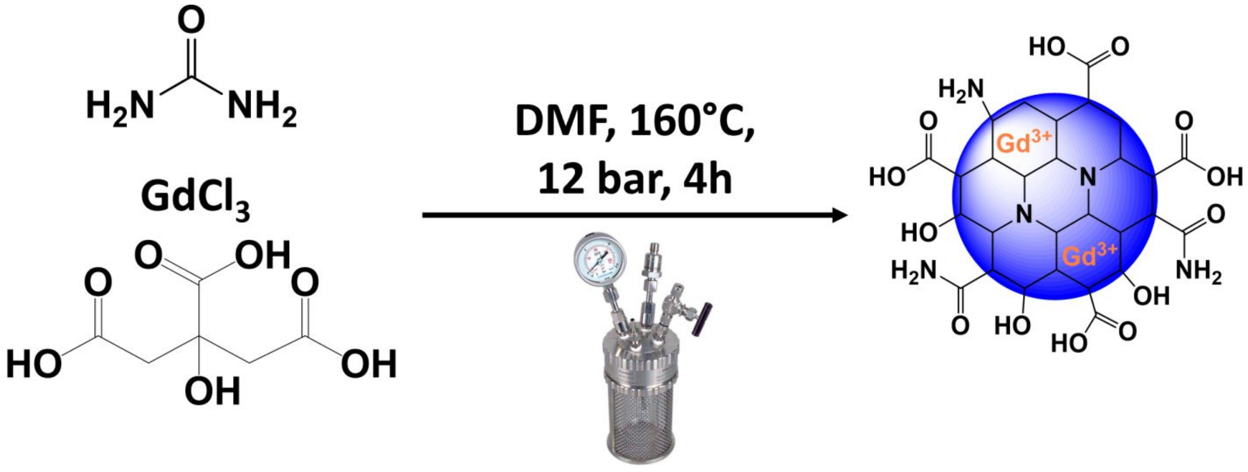

Figure S1. Schematic synthetic route for the synthesis of gadolinium-doped CDs.

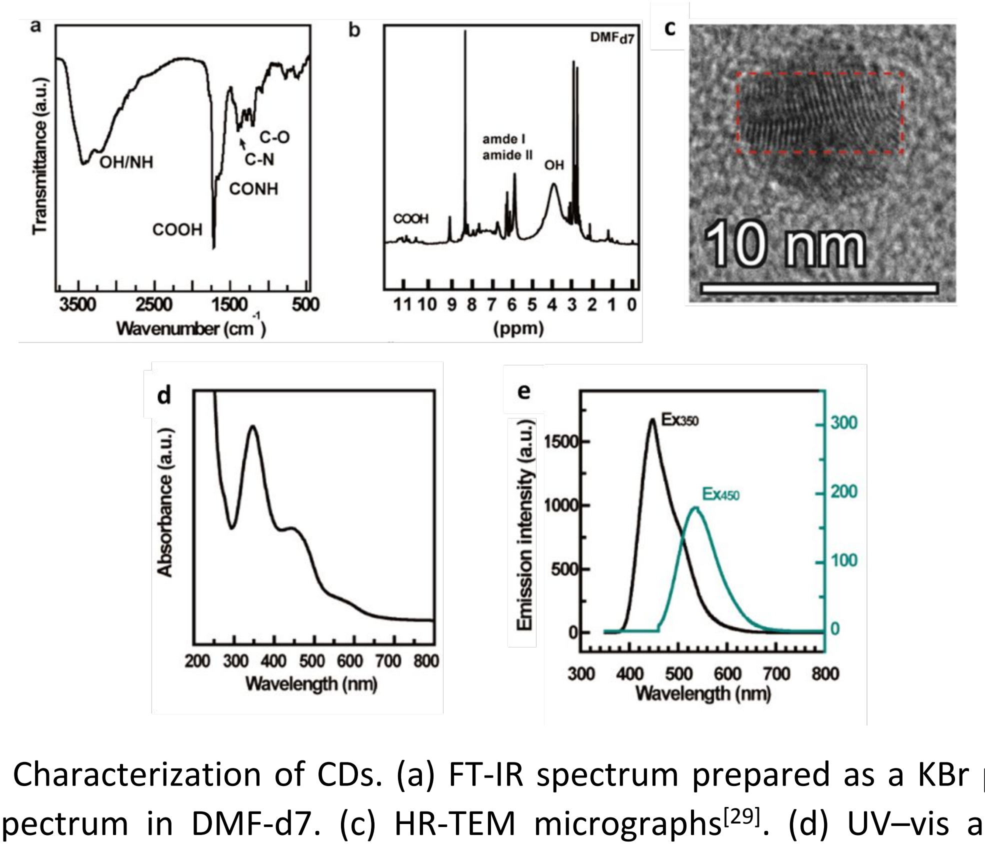

Figure S2. Characterization of CDs. (a) FT-IR spectrum prepared as a KBr pellet. (b) ^{1}H-NMR spectrum in DMF-d7. (c) HR-TEM micrographs[29]. (d) UV–vis absorption spectra in water dispersion. (e) 2D-emission spectra of aqueous solutions.

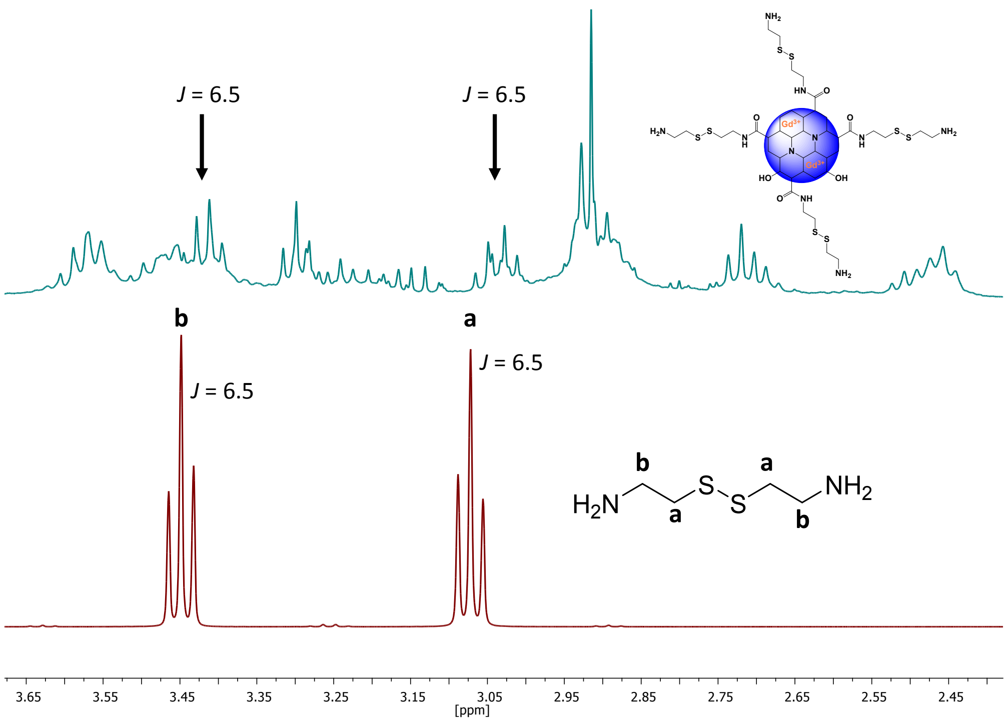

Figure S3. ^{1}H-NMR of CDs-Cys (D_2O)

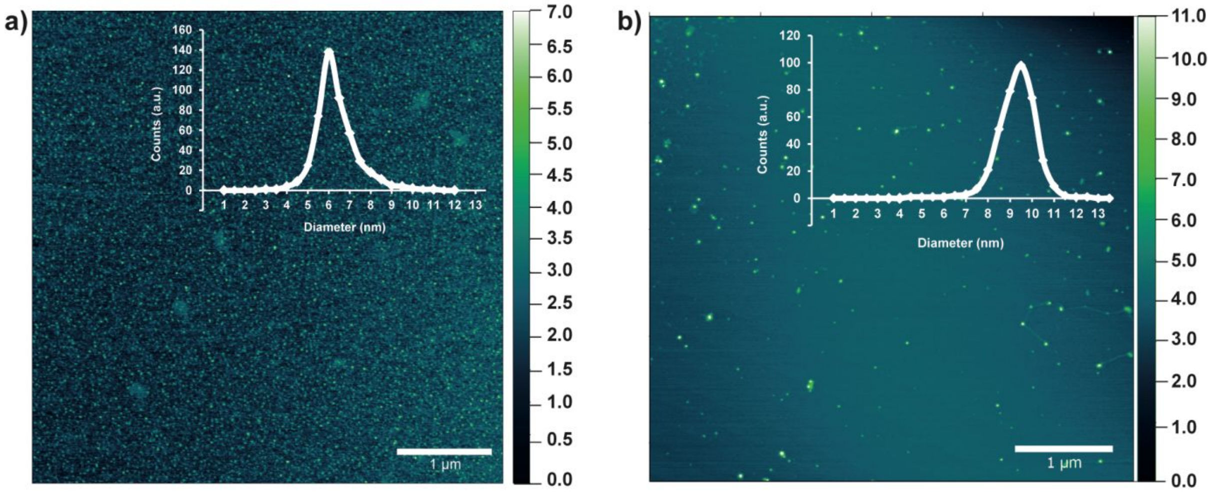

Figure S4. Atomic forse microscopy micrographs of CDs-Cyst (a) and CDs-Cyst-PAA$^{30:70}$ (b) obtained on MICA at a concentration of 0.1 mg mL^{-1}. Inserts: size distribution of the heights

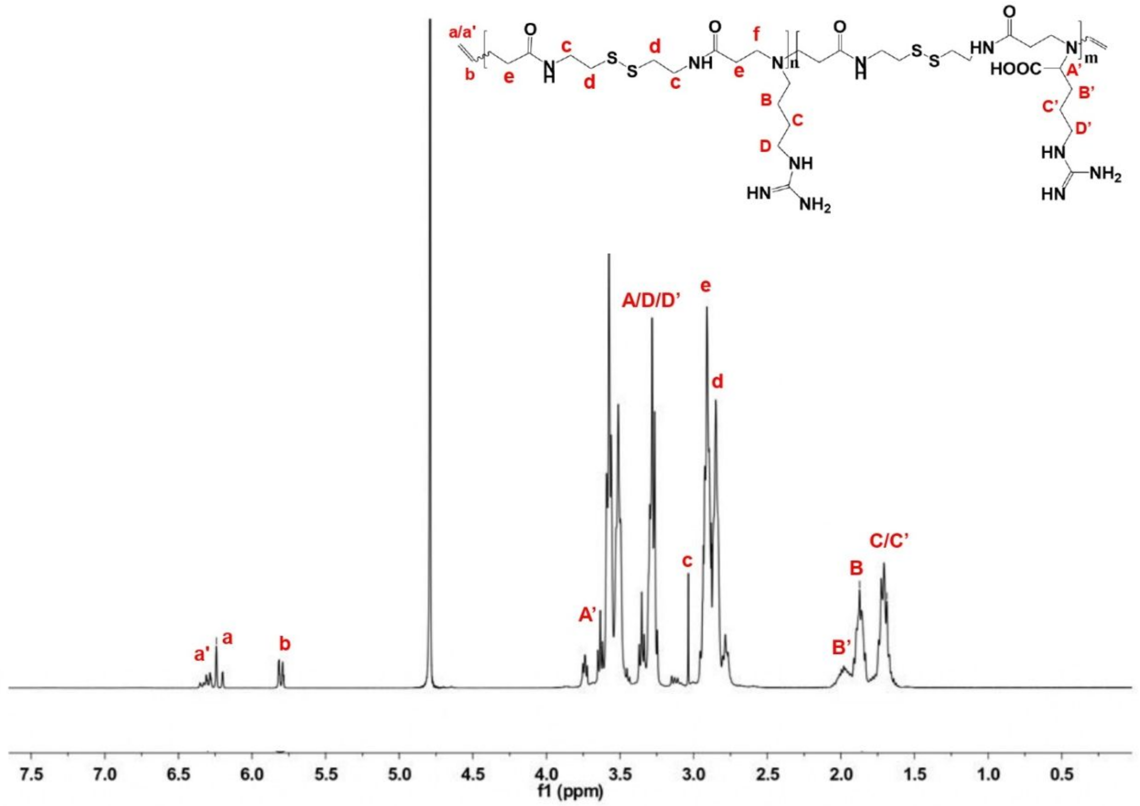

Figure S5. ^{1}H-NMR of PAA (D_2O)

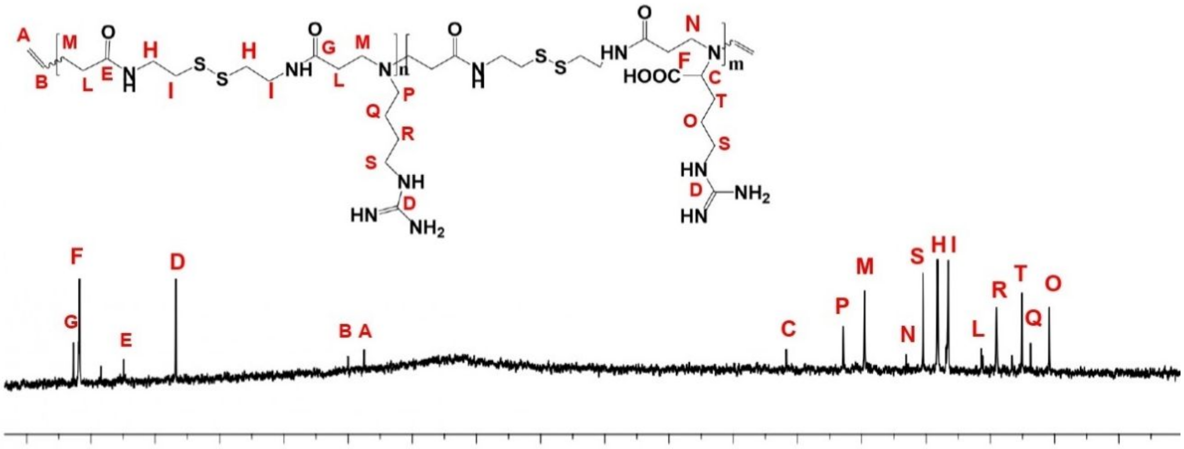

Figure S6. ^{13}C-NMR of PAA (D_2O)

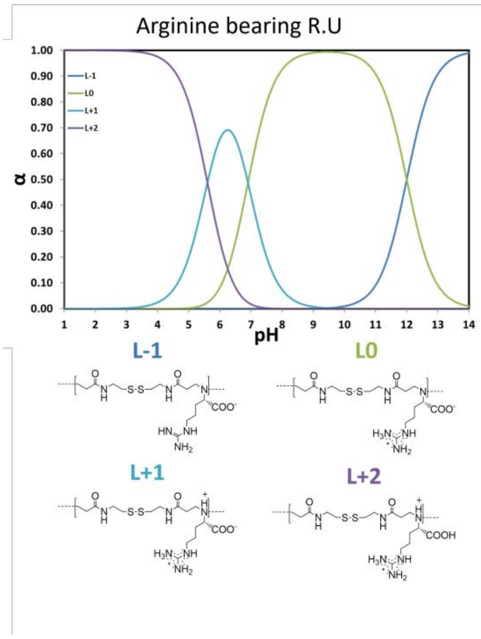

PAA[%Ag/%Arg]	Polymer net charge*	
	at pH 7.4	at pH 5.0
PAA[30/70]	+60	+186
PAA[50/50]	+84	+190
PAA[70/30]	+110	+194

*** In 100 repeat unit***

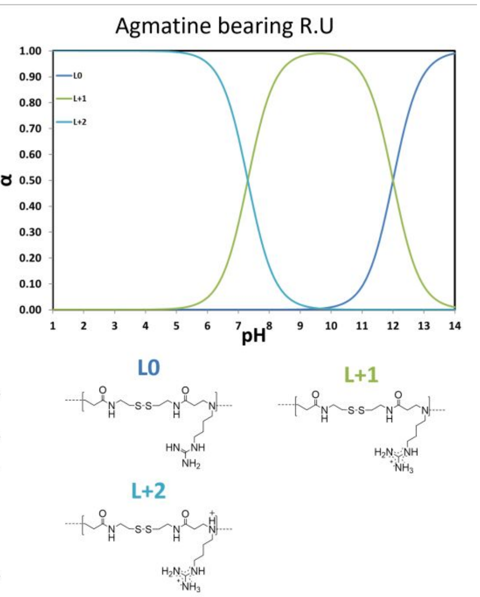

Figure S7. Speciation diagrams of PAA and different protonation state

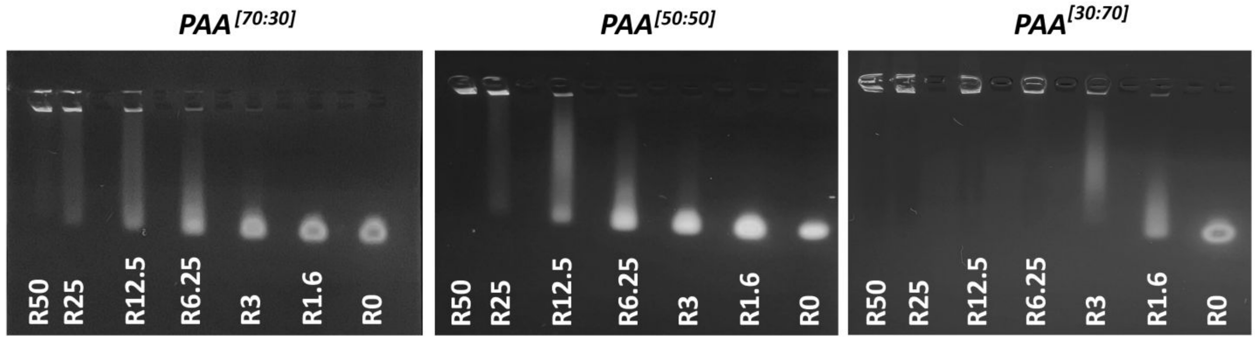

Figure S8. Electrophoretic runs at different PAA/siRNA weight ratios. Free siRNA was used as control (R0).

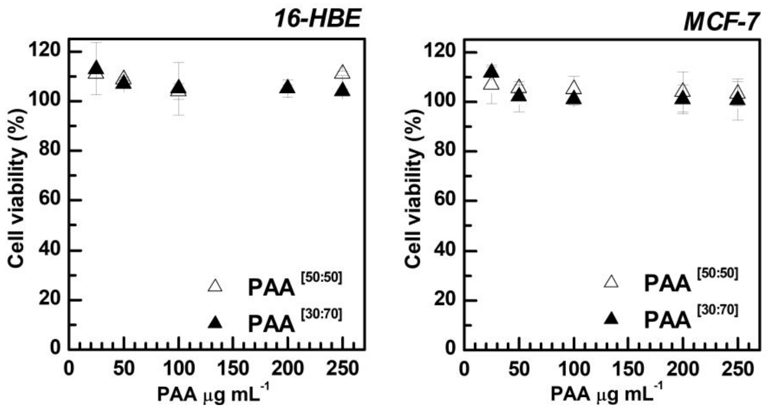

Figure S9. Cytocompatibility study of PAA[50:50] and PAA[30:70] on 16-HBE and MCF-7 after 24 h of incubation.

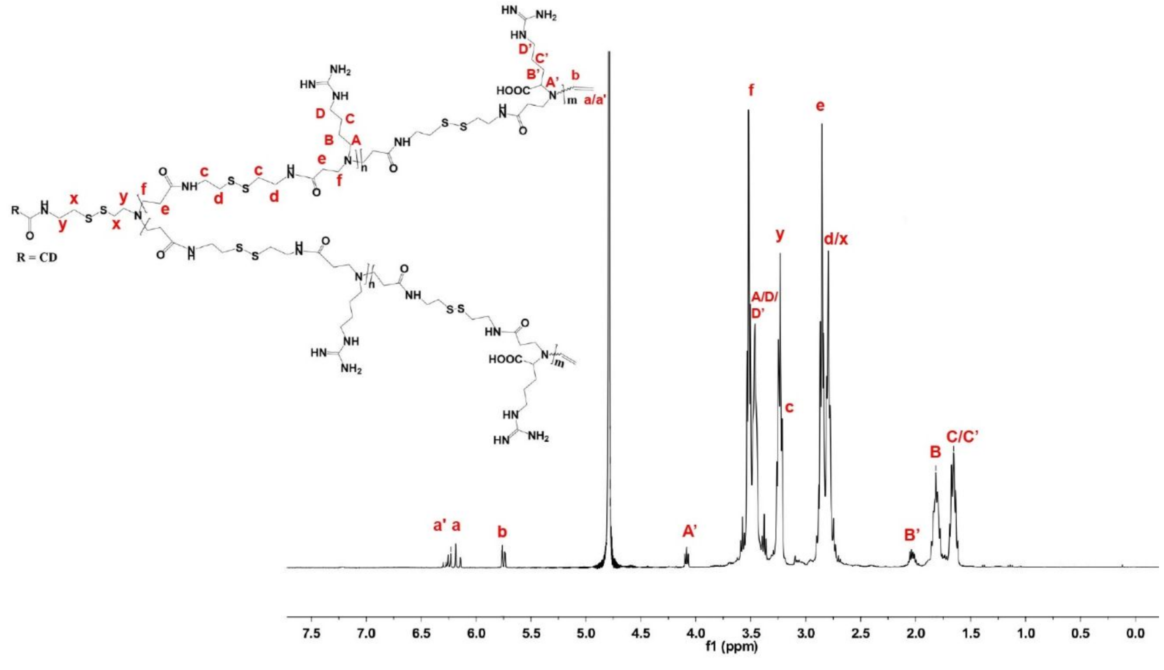

Figure S10 ^{1}H-NMR of the CDs-Cyst-PAA[30:70]conjugate (D_2O)

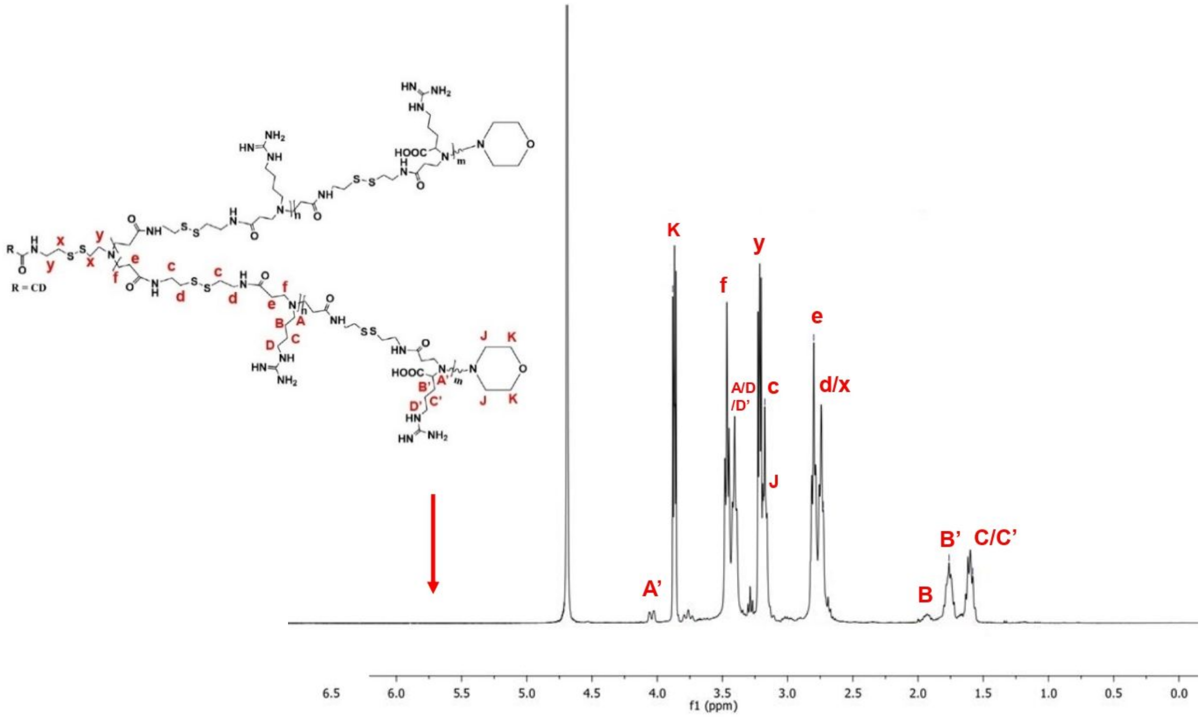

Figure S11 ^{1}H-NMR of the CDs-Cyst-PAA$^{[30:70]}$-M conjugate (D_2O)

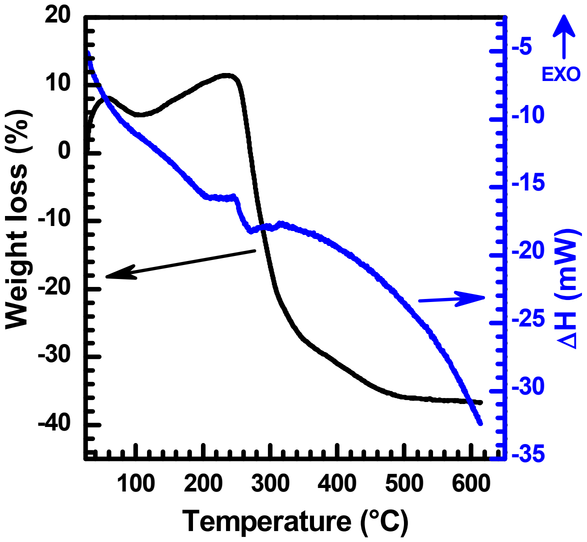

Figure S12. DSC-TGA of CDs-Cyst-PAA$^{[30:70]}$-M. The observable increment in apparent weight between 25 and 200 °C in the TGA curve can be ascribed to the oscillation of the crucible induced by the release of adsorbed gases and vapours; the evaporation

of free water is also visible in the DSC thermogram at 80-120 °C. Decomposition processes start at 200 °C, as suggested by the sharp weight loss accompanied with broad endothermal transitions.

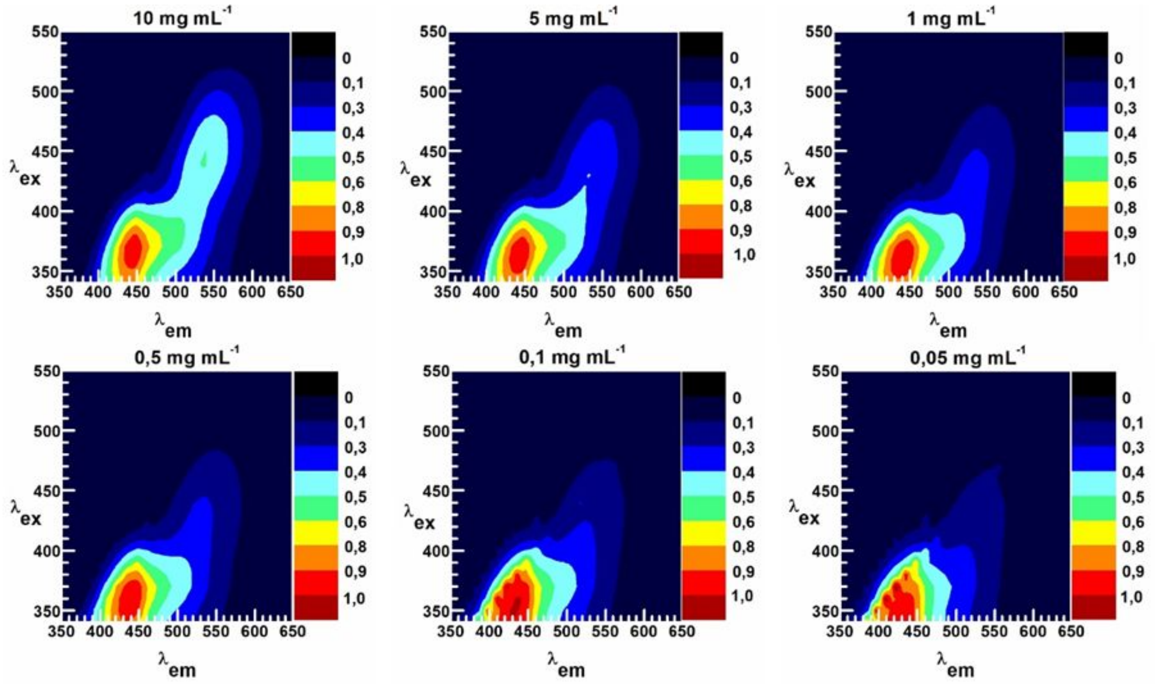

Figure S13. Normalized 3D fluorescence emission spectra of CDs-Cyst-PAA[30:70] -M at different concentration in artificial lysosomal fluid.
